# Enhancing cancer differentiation with synthetic MRI examinations via generative models: a systematic review

**DOI:** 10.1186/s13244-022-01315-3

**Published:** 2022-12-12

**Authors:** Avtantil Dimitriadis, Eleftherios Trivizakis, Nikolaos Papanikolaou, Manolis Tsiknakis, Kostas Marias

**Affiliations:** 1 Computational Biomedicine Laboratory (CBML), Foundation for Research and Technology Hellas (FORTH), 70013 Heraklion, Greece; 2 Department of Electrical and Computer Engineering, Hellenic Mediterranean University, 71410 Heraklion, Greece; 3 Medical School, University of Crete, 71003 Heraklion, Greece; 4 Computational Clinical Imaging Group, Centre of the Unknown, Champalimaud Foundation, 1400-038 Lisbon, Portugal; 5 The Royal Marsden NHS Foundation Trust, THe Institute of Cancer Research, London, UK

**Keywords:** Synthetic medical images, Data augmentation, Magnetic resonance imaging, Generative adversarial networks, Variational autoencoders

## Abstract

Contemporary deep learning-based decision systems are well-known for requiring high-volume datasets in order to produce generalized, reliable, and high-performing models. However, the collection of such datasets is challenging, requiring time-consuming processes involving also expert clinicians with limited time. In addition, data collection often raises ethical and legal issues and depends on costly and invasive procedures. Deep generative models such as generative adversarial networks and variational autoencoders can capture the underlying distribution of the examined data, allowing them to create new and unique instances of samples. This study aims to shed light on generative data augmentation techniques and corresponding best practices. Through in-depth investigation, we underline the limitations and potential methodology pitfalls from critical standpoint and aim to promote open science research by identifying publicly available open-source repositories and datasets.

## Key points

- Scarce and limited available data in oncology are burdensome for deep learning architectures, which tends to lead to poor decision systems.
- Heterogeneity in MRI images is a fundamental hurdle for generalization.
- Generative models are an emerging technology that could address these drawbacks through synthetic data augmentation.
- Evaluation metrics such as quantitative algorithms, qualitative assessment by experts, and a downstream task are essential for the validity of synthetic images.

## Introduction

Deep learning (DL) architectures gained immense popularity in the past few years and specifically when AlexNet [1] has shown outstanding performance and won the ImageNet competition [2] by a large margin compared to the then state-of-the-art machine learning models: however, the history of deep learning began several years ago. Initially, the inspiration emerged from the structure of the human brain. This led to artificial neural networks (ANN) designed for understanding the functionality of the brain [3] and inspired on conceptual level deep learning but not by attempting to match the low-level neural response itself [4]. From the 1940s to 1960s, ANN was known as cybernetics [5] where [6, 7] was the pioneers in developing theories of biological learning. Almost one decade after the implementation of the first analog model, the perceptron was introduced by Rosenblatt et al. [8] which could learn the weights for classifying samples based on previously seen examples from each category. The idea of using these networks became irrelevant for the next four decades, as ANN-based models could not successfully perform complex pattern recognition tasks using binary neurons. Nevertheless, the research continued, and when computers started to become fast enough, the idea of back-propagation using continuous neurons to operate floating-point multiplications emerged. Consequently, this led to the training of a neural network with one or two hidden layers [9, 10]. During the 1980s–1990s, the name switched to connectionism until 2006 when hardware advancements made feasible the stacking of more nonlinear layers and the term “deep” appeared and prevailed up to this day. Inspiration from neuroscience and specifically by the structure of the mammalian was critical one more time when the neocognitron [11] presented, an innovative and powerful architecture for image processing, which led [12] to the introduction of convolutional networks (ConvNets) featuring supervised learning algorithms such as back-propagation and end-to-end image analysis.

Applications of ConvNets in medical image analysis were first applied but with limited success in the early 1990s by [13, 14] mainly for detection of micro-calcifications in digital mammography and detection of lung nodules in chest radiographs [15]. Recently these models have been extended to a wide range of applications such as segmentation of nodules in computerized tomography (CT) images [16], organ segmentation [17], super-resolution [18], denoising [19] and cross-modality synthesis [20]. ConvNets are widely used deep architectures in the current state-of-the-art exploiting properties such as stationarity, locality and compositionality.

**Fig. 1:**
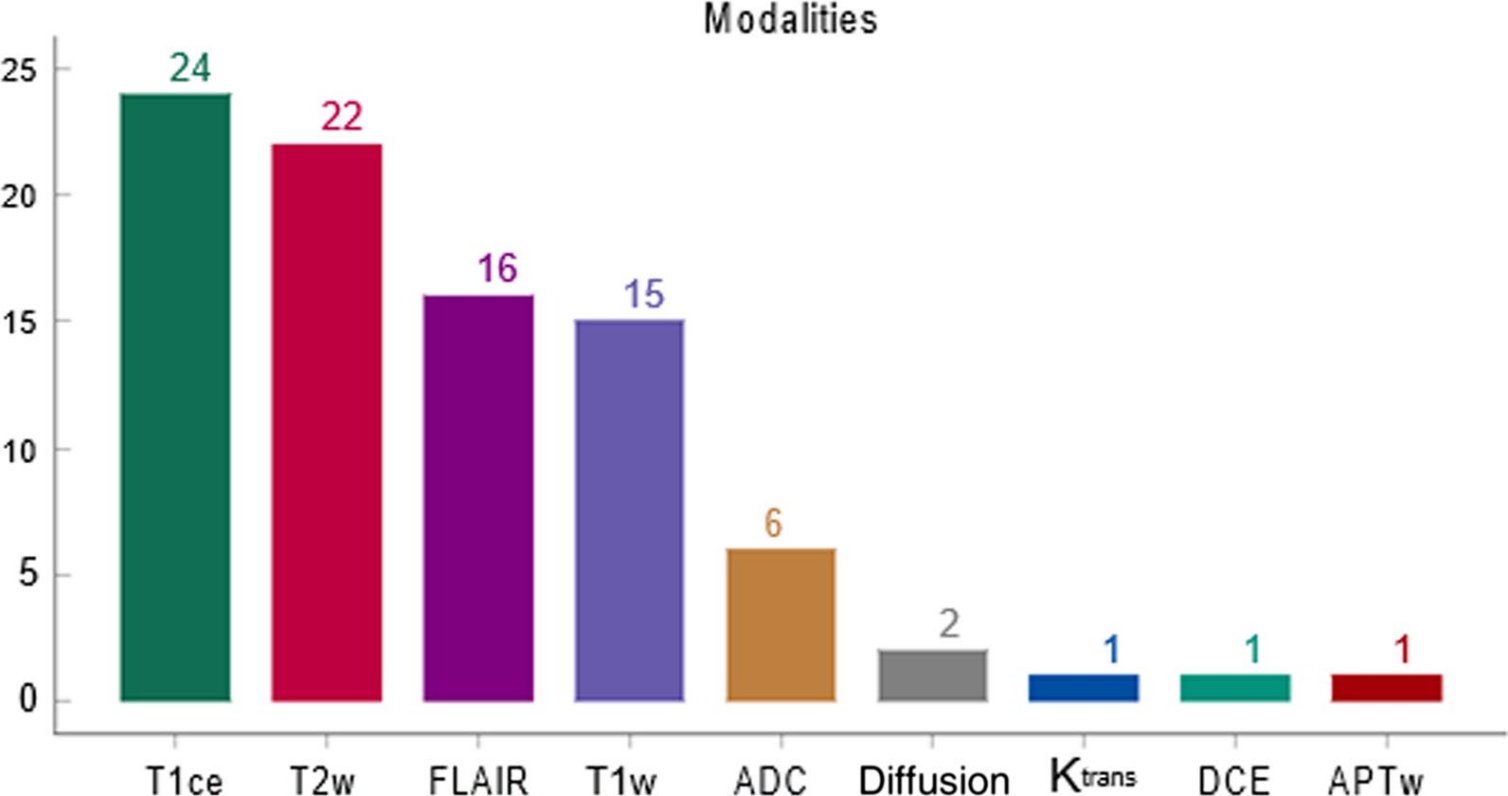
The MRI sequences were used in the examined studies. The brain anatomical region included the most studies which justify the reason that T1 contrast-enhanced (T1ce), T2-weighted (T2w), fluid-attenuated inversion recovery (FLAIR) and T1-weighted (T1w) modalities prevail in the above bar chart. Apparent diffusion coefficient, diffusion (ADC), $K^{trans}$, and dynamic contrast-enhanced (DCE) modalities were examined in the prostate anatomical region. The remained anatomical regions (i.e., pancreas, breast, liver) included as well the first four depicted modalities. Lastly, one study which concerned the brain region examined amide proton transfer weighted (APTw) modality

A key differentiating factor from other imaging domains is that magnetic resonance imaging (MRI) poses some significant challenges regarding the data collection because of the lack of tissue-specific values, different anatomical areas, varying imaging modalities (Fig. 1) different scanners and the absence of the imaging standardization across different vendors. The wide range of image acquisition protocols also contributes to the limited stability of deep models and can potentially be an impediment to a robust and generalized decision support system.

The scarcity of large and diverse patient cohorts with high-quality clinical data for specific clinical outcomes has been reported to be the most significant drawback of using DL in medical imaging tasks [21]. Data augmentation significantly enhances the convergence of DL models by synthetically generating new training samples. This can be achieved by incorporating trivial image processing techniques such as deformation, mirroring, flipping, zooming, cropping, rotating and other methods.

Unsupervised learning techniques such as generative adversarial networks (GANs) [22] and variational autoencoders [23] (VAE) have recently revolutionized data generation. A large number of images can be produced by a deep generative model from a random noise input or a binary segmentation mask. The GANs are usually comprised of two networks the generator *G* that creates new samples from noise and the discriminator *D* that distinguishes among the valid and invalid synthetic samples. The convergence of these models is achieved simultaneously by an algorithm that is based on game theory with a minimax loss. VAEs are commonly used for feature extraction by compressing the input to a compact representation, but they have lately been employed for generative properties by manipulating the latent space.

Generation of synthetic data for cross-sectional imaging modalities in oncology is highly challenging since there is a significant underlying biological variability that leads to multiple phenotypic and genetic subtypes of cancerous tumors [24]. Additionally, in this review the focus on MRI was decided because of the unique properties and limitations of this modality, such as the lack of measured signals, dependence on the scanner vendor, acquisition parameters and image acquisition protocols. It is argued that a generalized generative model could potentially overcome some of these drawbacks by enhancing the diversity and size of the examined patient cohort. A few other reviews of GANs for various medical applications, including generating images, have been published. Singh et al. [25] focused mostly on the general technical attributes of GANs. Sorin et al. [26] reported general radiology applications such as reconstruction, denoising, generation, cross-modality translation and segmentation. Yi et al. [27] presented studies with GANs for varying imaging and clinical applications of medical imaging in general. Additionally, a preprint review [28] for GANs that extends the existing reviews by including patient privacy and lesion progression monitoring applications in generative cancer imaging was explored. Notably, Wei et al. [29] reported on VAE-based applications in biomedical informatics, including data generation.

The current literature review presents from a critical standpoint studies that implement novel deep generative data augmentation techniques on cancer MRI examinations, aiming to highlight the best techniques for synthetic tumor representations, identify best practices for data analysis, promote open science approaches based on widely available public data and open-access source code repositories for improved reproducibility. Another key element of this study is to report the most robust evaluation techniques of the generated samples, which include a visual assessment by experts, direct quantitative metrics prior to analysis and indirect methods from the performance enhancements of the downstream analysis.

The sections of the rest of the review are organized as follows: in sect. 2  the search methodology is presented, in sect. 3  key generative architectures used by the examined studies are analyzed, in sect. 4  details about the studies included in this review are reported, in sect. 5  the adopted evaluation methods, limitations and future remarks are discussed, and the final section concludes the review.

## Search methodology

### Search strategy

A systematic review of studies that use data augmentation techniques that enhance cancer differentiation via deep generative models was performed to assess the impact of GANs and VAE applications in oncology. This systematic review was conducted by following the reporting checklist of the Preferred Reporting Items for Reviews and Meta-Analyses (PRISMA) [30]. For the purpose of this study, a comprehensive literature search was undertaken to identify research papers employing a deep generative model to synthesize cancer MRI images. A protocol was developed in advance to document the analysis method and inclusion criteria. We utilized Scopus, PubMed, Google Scholar, IEEE, Web of Science and Arxiv websites. The papers were selected by querying Scopus and PubMed on peer-reviewed journals and conference/proceeding publications between January 1, 2017, and June 30, 2021. The query contained ((“generative” “adversarial” “networks”) OR (“GANs”) OR (“Variational” “auto” “encoders”) OR (“VAE”)) AND ((“magnetic” “resonance” “imaging”) OR (“MRI”) ) AND (“data” “augmentation”) OR (“oncology” AND “synthe*”). Additionally, on the remaining websites, key words such as “GANs” or “VAE,” “data” “augmentation,” “synthetic” “MRI” “examination” “generation,” “oncology” were used.

### Study selection

**Fig. 2:**
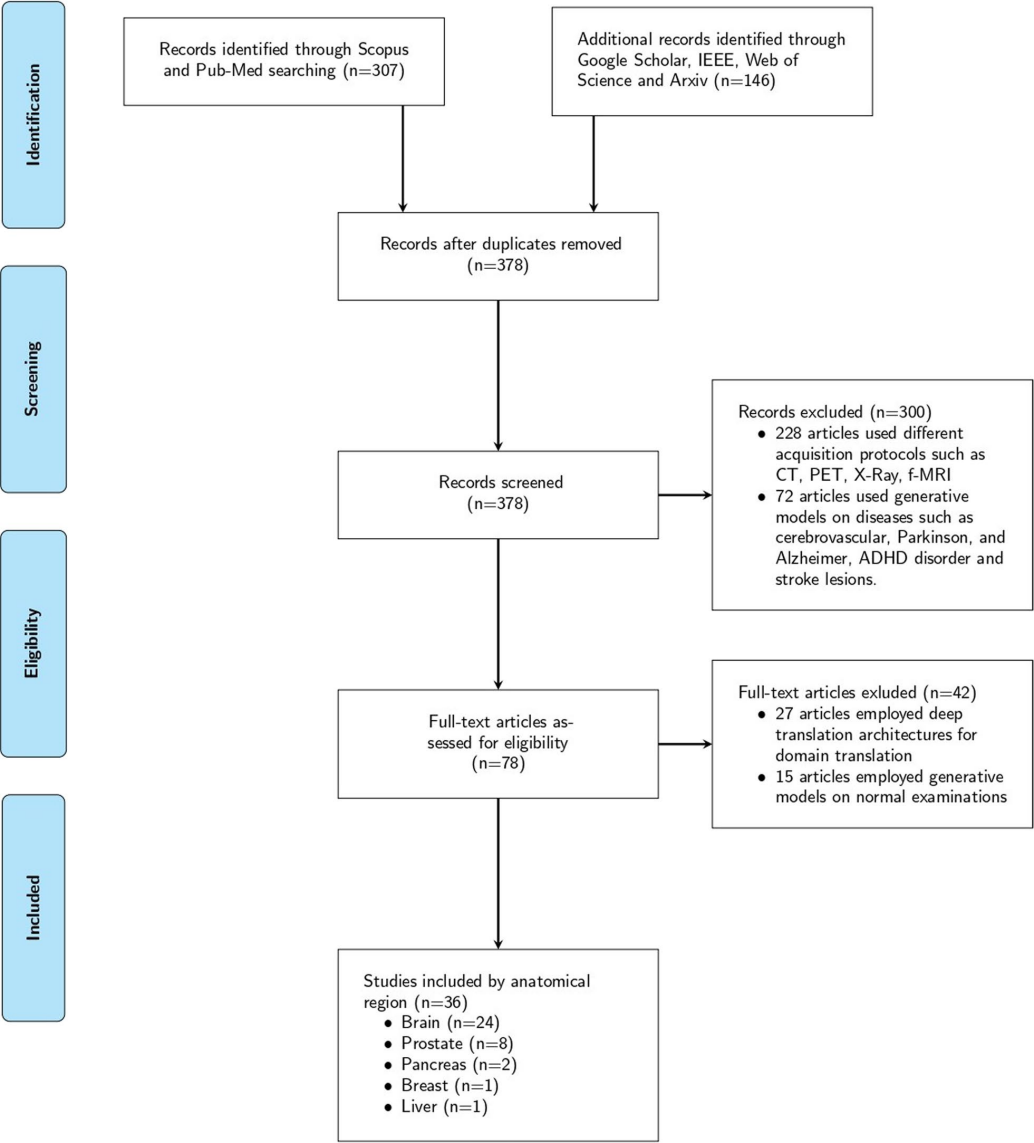
The PRISMA flow diagram for the followed search methodology

**Fig. 3:**
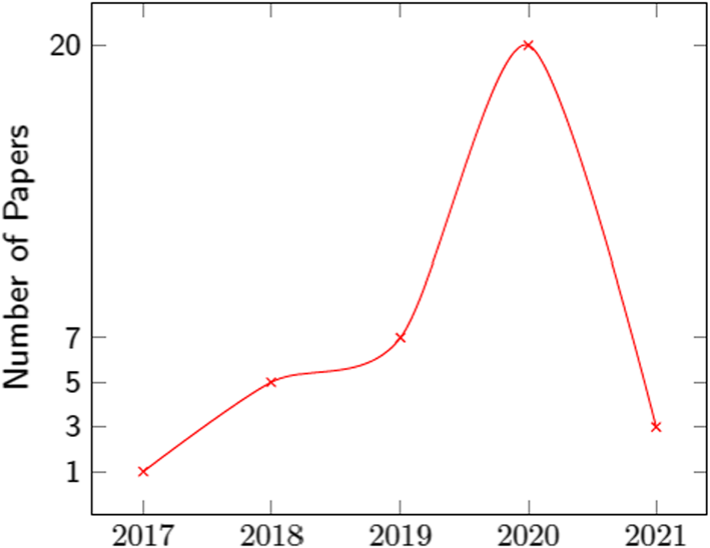
The graph depicts the gradual increase of studies on data augmentation with synthetic MRI examinations to enhance cancer differentiation. In the year 2020, the number of studies exceeded the total number of the previous three years indicating the interest of researchers in the field

Two reviewers screened the titles, abstracts and conclusions of the records independently and papers that were clearly not related to the subject matter were discarded. During the first screening phase, the abstracts and conclusions of papers were carefully assessed. Despite the fact that the majority of these papers contained a subset of the searched keywords, a second full-text screening showed that the methodology presented in many of these works was not relevant to deep learning architectures for data augmentation, and thus they were removed. In total, the current study reviewed 36 papers that were based either on widely used, open datasets or on custom in-house data. Most of the custom datasets had data from patients with neoplasms that had been verified by a biopsy, and one study was comprised of data from patients who had either a biopsy or a follow-up imaging examination within the past twelve months. Notably, these papers included different imaging protocols as depicted in Fig. 1. The study selection process is summarized in Fig. 2, and the publishing timeline of the examined studies is presented in Fig. 3.

### Risk of bias assessment

**Fig. 4:**
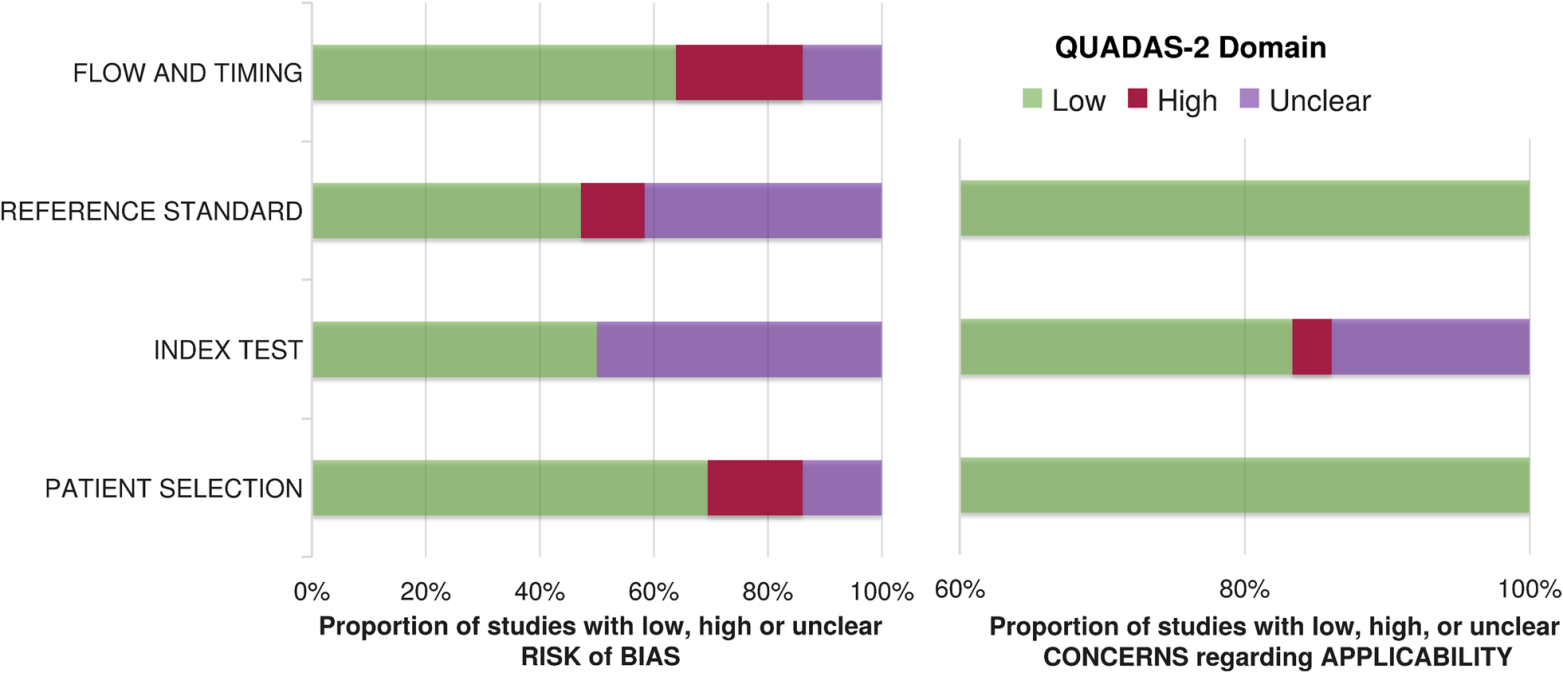
Summary results of QUADAS-2 tool on risk of bias and applicability concerns for the included studies in the present systematic review

The updated QUADAS-2 (Quality Assessment of Diagnostic Accuracy Studies-2) [31] criteria were employed by the two reviewers to evaluate the risk of bias and applicability of the included studies. Each item was rated as “low,” “high” or “unclear.” The item was scored as “unclear” when the absolute information was not provided or was insufficient to permit a judgment. The results of bias risk and applicability are summarized in Fig. 4.

## Generative models

### Noise to image

**Fig. 5:**
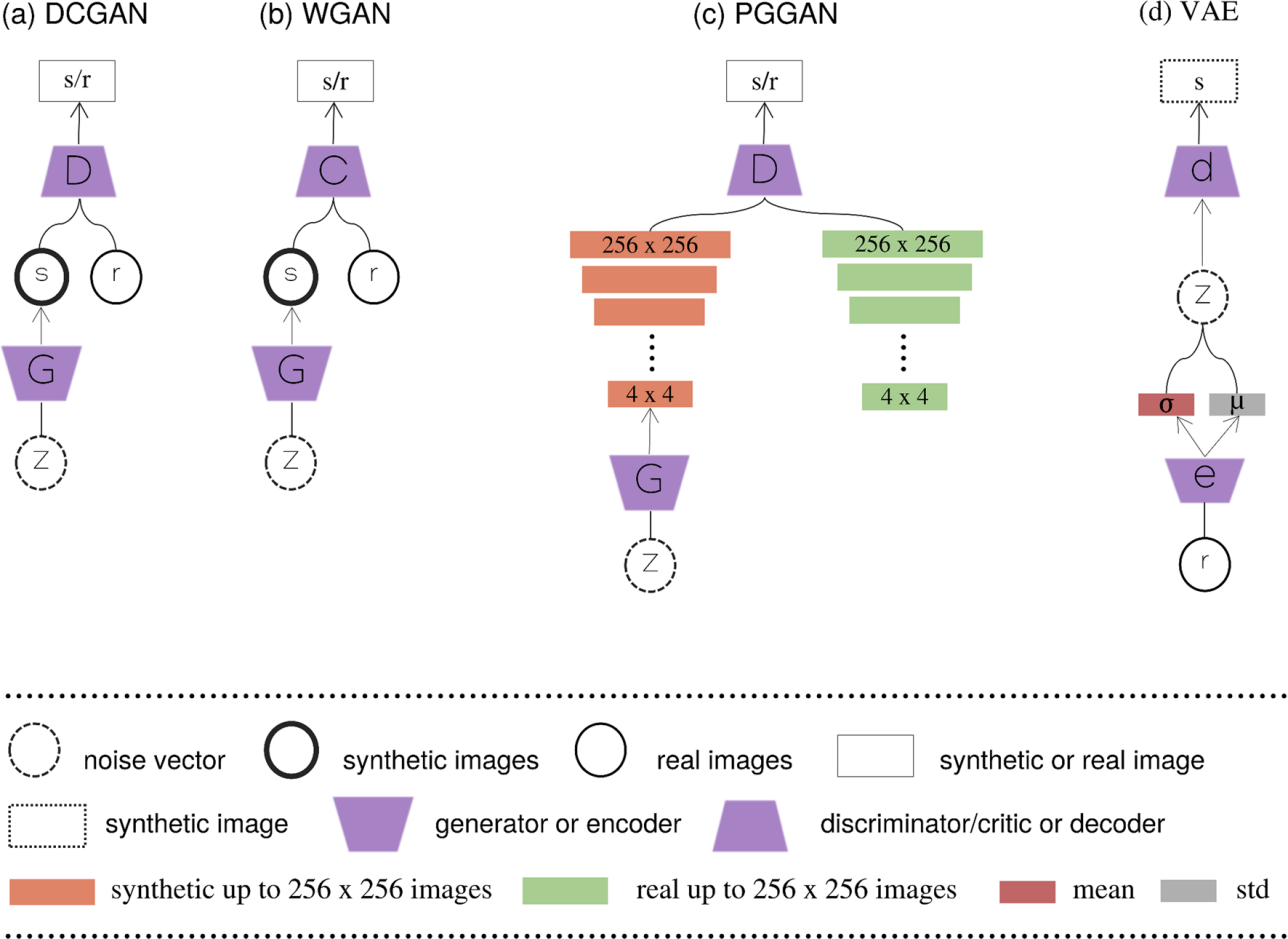
A schematic view of variants of GANs and VAE. **a** The primary idea of the DCGAN compared to vanilla GAN is that adds transposed convolutional layers between the input vector Z and the output image in the generator. In addition, the discriminator incorporates convolutional layers to classify the generated and real images with the corresponding label real or synthetic. **b** Training a GAN is not trivial. Such models may never converge and issues such as model collapses and vanishing of gradients are common. WGAN proposes a new cost function using Wasserstein distance that has a smoother gradient. The discriminator is referred to as the critic who returns a value in a range, instead of 0 or 1, and therefore acts less strictly. **c** The training in PGGAN starts with a single convolution block in both generator and discriminator leading to 4 x 4 synthetic images. Real images are downsampled also to be of size 4 x 4. After a few iterations, another layer of convolution is introduced in both networks until desired resolution (e.g., 256 x 256 in the schematic). By progressively growing the network learns high-level structures first followed by finer-scale details available at higher resolutions. **d** In contrast to traditional autoencoders, VAE is both probabilistic and generative. The encoder learns the mean codings, $\mu$, and standard deviation codings, $\sigma$. Therefore the model is capable of randomly sample from a Gaussian distribution and generating the latent variables Z. These latent variables are then “decoded” to reconstruct the input

**Fig. 6:**
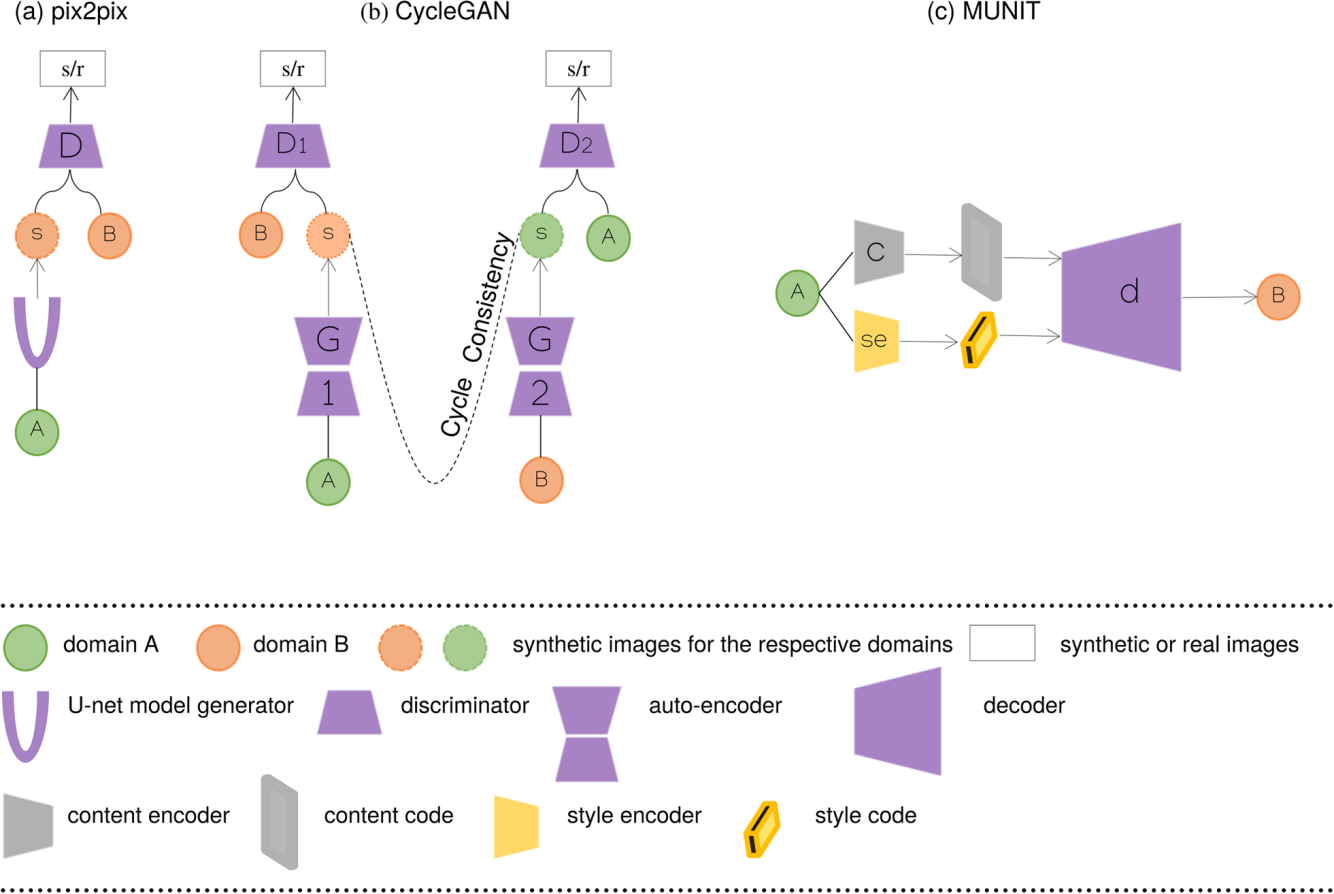
Generative architectures for image-to-image translation. **a** The pix2pix is an extension of the conditional GAN architecture that provides control over the generated image. The U-net model generator translates images from one domain to another and through skip connections the low-level features are shared. The discriminator judges whether a patch of an image is real or synthetic instead of judging the whole image, while the modified loss function allows the generated image to be plausible in the content of the target domain. **b** CycleGAN is designed specifically to perform image-to-image translation on unpaired sets of images. The architecture uses two generators and two discriminators. The two generators are often variations of autoencoders where they take as input an image and output an image as output; the discriminator, however, takes as input an image and outputs one single value. In the case of CycleGAN, a generator gets further feedback from the other generator. This feedback confirms whether an image generated by a generator is cycle consistent, meaning that applying successively both generators on an image should produce a similar image. **c** In the MUNIT architecture, the image representation is decomposed into a content code and style code through the respective encoders. The content code and style code is recombined to translate an image to the target domain. By sampling different style codes the model is capable of producing diverse and multimodal outputs

Formulating the objective loss function of a GAN model leads the researchers to create a plethora of such models (Fig. 5, Fig. 6) with different kinds of structures and for a variety of objectives. Deep Convolutional Generative Adversarial Networks (DCGAN) (Fig. 5 a) [32] include similar to Vanilla GAN [22] a generator and discriminator and ,nevertheless, instead of a fully connected layer, incorporates a fully convolutional layer which produces improved synthetic images and stabilizes the training process. Likewise, Batch Normalization and LeakyReLU activation function were two important modifications. As an alternative, Wasserstein GAN [33] (WGAN) (Fig. 5 b) replaced the discriminator with a critic where instead of predicting the probability of synthetic images as being real or fake, scores regarding the realness or fakeness of a given image are provided. The generator is trained by minimization of the distance between the distribution of real and generated examples. Progressive Growing of GANs (PGGANs) (Fig. 5 c) introduced by Karras et al. [34] to improve quality, stability and variation. The rationale of this approximation is to progressively increase the generator and discriminator, which starts from a low resolution and adds new layers, whereas the training progresses. Variational autoencoders (VAEs) (Fig. 5 d) [23] are another variant of autoencoder where the network through mapping the input into distribution in the latent space with the encoder network, enables the ability to sample first the latent vector from the distribution and then using the decoder to generate new data.

### Image to image

**Fig. 7:**
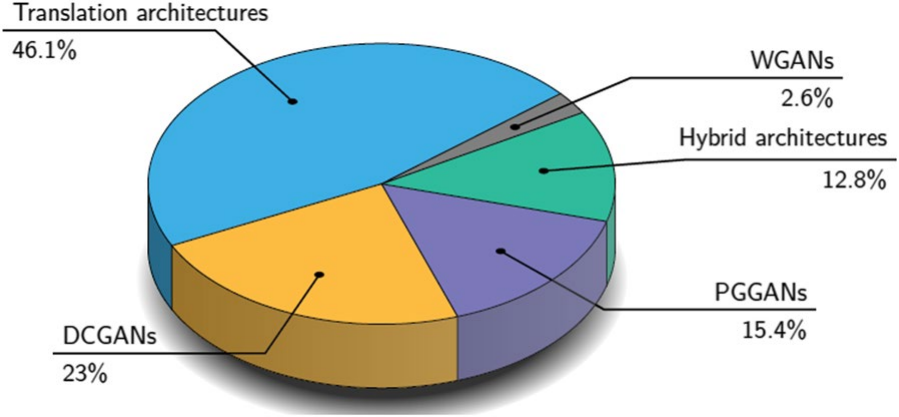
The family of architectures proposed in the examined studies. WGANs, Wasserstein generative adversarial networks; PGGANs, progressive growing of generative adversarial networks; DCGANs, deep convolutional generative adversarial networks. Almost half of the examined studies employed translation architectures (i.e., pix2pix, cycleGAN, MUNIT) to translate from one MRI sequence to another or to incorporate different types of lesions into a healthy subject. The hybrid architectures consist of the combination of GANs and VAE to increase the stability of the training and to generate higher-quality synthetic images. The studies with the remained architectures focused on generating MRI images from a noise vector

The pix2pix GAN [35] (Fig. 6 a) is a supervised image-to-image model, where a target image is synthesized conditional on a given input image. The cyclic adversarial generative network (CycleGAN) (Fig. 6 b) [36] is composed of two generators and two discriminators to perform higher-resolution image-to-image translation using unpaired data. Multimodal Unsupervised Image-to-Image Translation [37] (MUNIT) (Fig. 6 c) architecture trains two auto-encoders, one to encode the content and the other to encode the style of images. Furthermore, the image representation can be decomposed into a content code that is domain-invariant, and a style code that captures domain-specific properties. The architecture recombines the content code with a random style code sampled from the style space of the target domain, to translate an image to another domain. Figure 7 summarizes the previously mentioned architectures proposed in the examined studies. The category of “Translation architectures” includes mainly the pix2pix, CycleGAN and MUNIT architectures, whereas the “hybrid architectures” are when GANs and VAEs are combined.

## Deep generative models by anatomical region

### Brain

Beers et al. [38] were the pioneers who employed PGGANs on retinal fundus images and brain tumor multi-modal MRI images. The generative network was able to synthesize high-resolution medical images that were both realistic and phenotypically diverse. The authors concatenated the segmentation glioma maps along with multi-modal MRI images as color channels, to allow the network to synthesize anatomically correct tumor structures in the synthetic MRI slices.

**Fig. 8:**
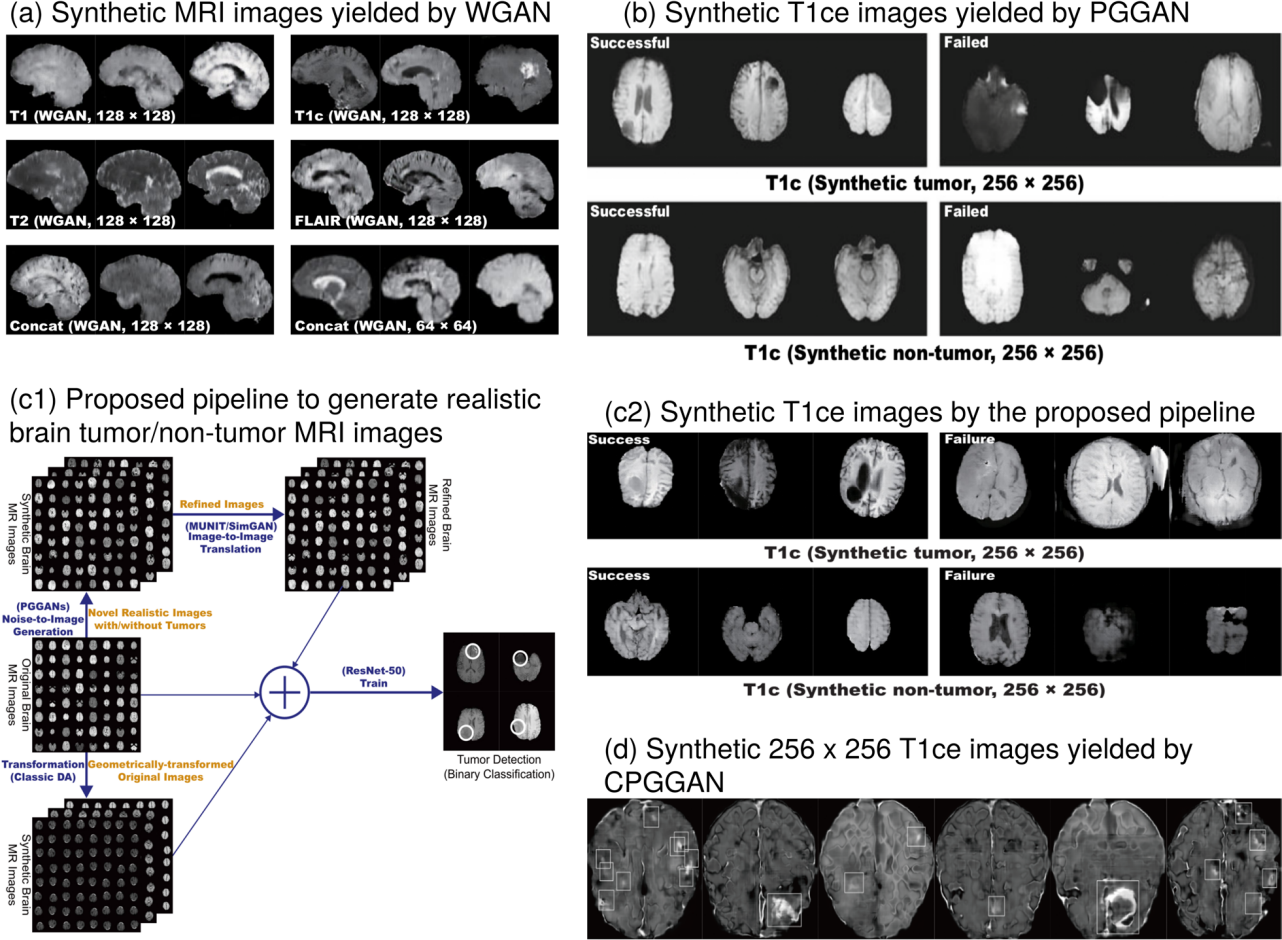
Key findings and a proposed pipeline by the examined studies. **a** Depicts the synthetic samples in each MRI sequence [39]. **b** Example of T1 contrast-enhanced synthetic tumor and normal examinations in both successful and failed cases [41]. **c1** The proposed noise-to-image and image-to-image combined architectures for tumor detection [42]. **c2** Example of T1 contrast-enhanced synthetic tumor and normal examinations in both successful and failed cases [42]. **d** Synthetic T1 contrast-enhanced samples with the tumor bounding boxes [44]. By “non-tumor” areas the authors refer to “normal examinations”

Han et al. [39] proposed a methodology for addressing the tumor diversity in generated medical images with realistic morphological characteristics. Thus, they compared two generative architectures, DCGAN and WGAN for the sake of this objective. The latter architecture produced synthetic images with increased tumor diversity in multi-sequence MRI, and it was successfully captured the sequence-specific texture and tumor appearance (Fig. 8 a). In addition, an expert physician evaluated via Visual Turing Test [40] all the generated images derived from WGAN as more realistic. Employing different types of generative models such as PGGAN and with the aim to synthesize a single image type (Fig. 8 b) Han et al. [41] adopted these multi-stage generative architectures to augment the original dataset and perform a downstream task. In another work, Han et al. [42] implemented a noise-to-image and image-to-image adversarial architecture (Fig. 8 c1) for sample generation (Fig. 8 c2) to increase the performance of the classification in terms of accuracy, sensitivity and specificity. Additionally, the proposed pipeline was divided into a two-stage approach to improve model convergence. Initially, the PGGAN generates the high-resolution MRI images and two translation frameworks MUNIT [37] and SimGAN [43] were used consecutively to refine the synthetic images and increase their realism, and anatomical diversity. The combination of traditional data augmented techniques with the refined images increased the performance of the downstream task and improved tumor detection. The Conditional Progressive Growing of GANs (CPGGAN) [44] is an expansion of a previous architecture [41] and is conditioned to generate MRI images with brain metastases at specific positions and sizes (Fig. 8 d) since brain metastases are the most common intracranial tumors, getting prevalent as oncological treatments ameliorate cancer patients’ survival [45].

Shin et al. [46] used the pix2pix GAN model to generate synthetic MRI images with brain tumors. At first, the model was trained to segment normal brain anatomy from the T1-weighted images of the ADNI dataset. The same model is used again on different MRI sequences of the BRATS dataset. Combining brain anatomy and tumor segmentation, the overall segmentation of the brain with tumor is acquired. The authors used the segmentation masks and the BRATS dataset to generate the synthetic brain MRIs with lesions in four different sequences. Furthermore, by adjusting these masks (e.g., shifting tumor size, changing tumor location or locating tumor on an otherwise tumor-free brain label), they introduced variability in the anatomical regions and tumor characteristics. Lastly, when the original dataset augmented with synthetic images. the results of the downstream task revealed a performance that outperformed compared to the model trained with only real data.

**Fig. 9:**
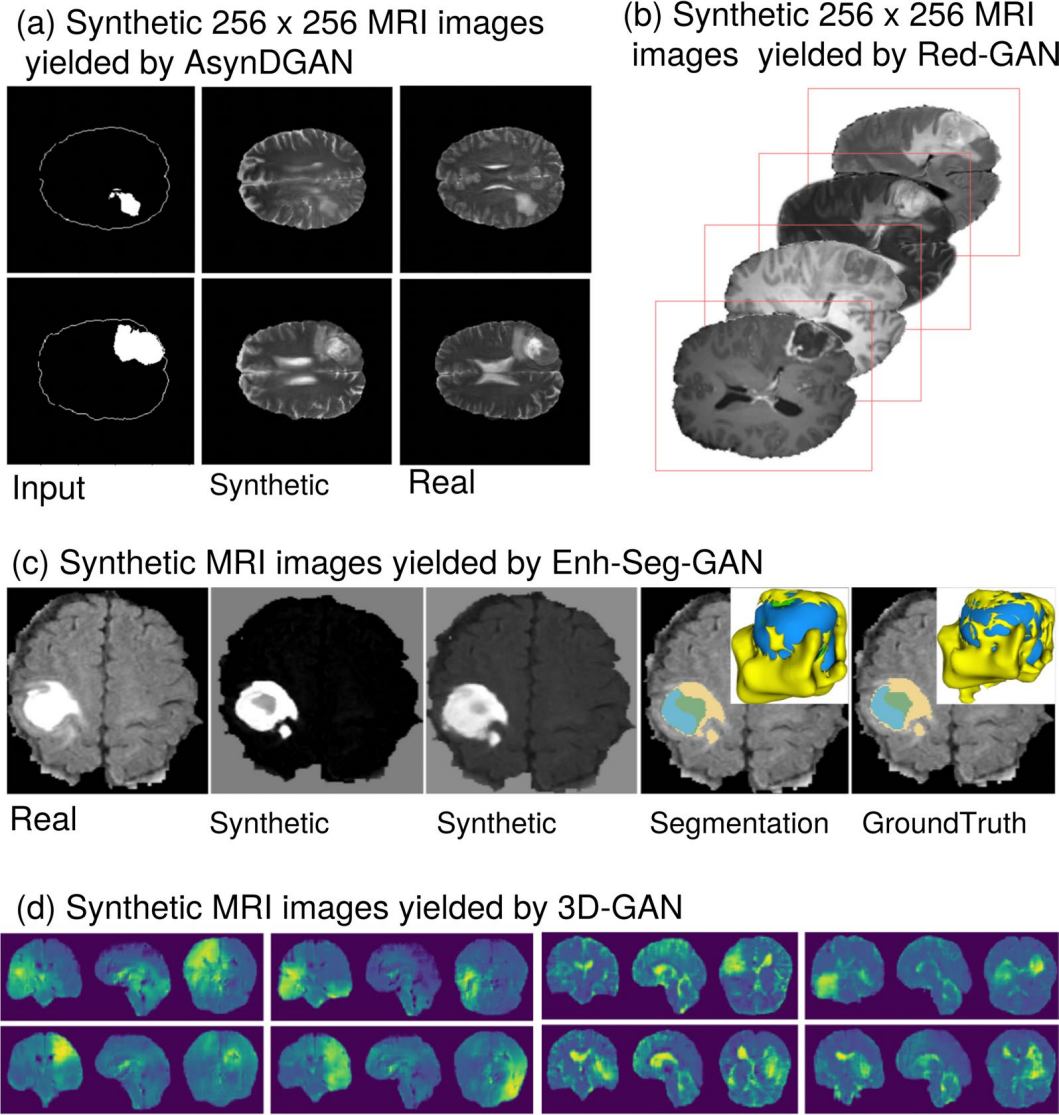
Key generated samples for the examined studies. **a** The input of the AsynDGAN network, the generated sample and the corresponding real image [47, 48]. **b** Generated images conditioned on lesion masks [50]. **c** An example of generated images with the corresponding segmentation and groundtruth. The colors mean yellow: edema, blue: non-enhancing, and green: enhancing tumor. The 3D representation of the tumor is presented on the top right [56]. **d** Synthetic samples of severe cases of brain tumor, for better visualization the authors displayed color-mapped images where yellow indicates higher and blue indicates lower intensity [52]

The Asynchronized Discriminator GAN (AsynDGAN [47, 48]) is a distributed learning framework that is comprised of a 9-block ResNet auto-encoder (generator) and various PatchGANs [35] (multiple discriminators) that can capture the localized anatomy of real and synthetic data (Fig. 9 a). The centralized generator learns the joint distribution of multiple data from different institutions where for each institution exist a discriminator to classify the local real data and the synthetic data. Thus, the framework ensures that the generated images can be shared across multiple institutes with no privacy concerns and promote collaborative research. The performance of a downstream task such as brain tumor segmentation, suggests that models trained with ASynDGAN synthetic data achieved close-to-real performance when compared to models trained entirely on real data.

Medical imaging datasets are frequently limited in terms of size and diversity, especially in oncology since the natural prevalence of the disease leads to imbalanced sets. Deepak et al. [49] applied a multi-scale gradient GAN (MSG-GAN) due to simplicity and robustness compared to DCGAN and PGGAN, to generate meningioma tumor MRI samples in coronal plane. The progressively growing generator accomplished to augment the class imbalanced dataset by 55 samples, improving the balanced accuracy score up-to 93%. Likewise, Qasim et al. [50] modified SPADE-GAN [51] and proposed Red-GAN, by introducing an adversarial pipeline conditioned on both local and global information. The 7-SPADE ResNet as a generator synthesized MRI images across multiple modalities from lesion masks (Fig. 9 b) and then together with real images was separately given as input to the U-Net segmentation model to ensure that synthetic and real images are in close proximity in the latent representation. The feature representations were obtained from the U-Net along with lesion masks, and the corresponding synthetic or real slices were fed as input to the PatchGAN discriminator. Dice performance when the downstream model trained only on synthetic images reached 0.659, while an increase up to 5% was achieved for most of the sparse classes when the original dataset was augmented with synthetic samples.

VAE is stable during the training process, but blurry images can be produced. On the other hand, GANs can synthesize realistic images, but they are unstable during training. Kwon et al. [52] proposed a 3D-GAN model that leverages the $\alpha$-GAN [53] architecture which essentially combines the advantages of the aforementioned networks with an additional auto-encoder and a code discriminator on the top of the existing generator and discriminator. Moreover, the Wasserstein distance with gradient penalty was introduced to reduce the training instability. The authors claim that the model generates realistic samples with brain tumor lesions at various positions while properly reacting the characteristics of different modalities (Fig. 9 d). By using principal component analysis (PCA) cluster representation, it was demonstrated that a moderate larger latent noise vector assists the model to escape from model collapse.

The combination of auto-encoders and GANs [54] was proposed as a hybrid GAN framework to improve diversity in local areas of MRI images and augment the available samples in the examined dataset. Initially, real MRI images are divided into equal-sized patches and fed as input to the encoder–decoder module where synthetic patches can be sampled. Next, the generated patches along with a constrained noise vector are set into RU-NET generator, where finally fake patches are integrated into full-sized synthetic images. Notably, binary classification (i.e., tumor, non-tumor) performance decreased when combining synthetic with real data, while the accuracy reached at highest when the model trained only on synthetic data. Pesteie et al. [55] proposed a conditional VAE fitted with a novel adaptive training algorithm. This technique was applied in ultrasound and MRI data for sample generation in segmentation tasks. The examinations, annotations and latent variables were kept, independent. The model learns to synthesize data from joint distribution composed of a random latent sample and an encoded segmentation mask. Additionally, two augmentation techniques were employed with a static and a trainable adaptive parameter for deforming the input segmentation mask.

Lesion segmentation in medical images is a challenging task and can be achieved by automated or semi-automated detection of lesions or organs within 2D or 3D examinations. The high variability of tumors in terms of shape and texture is the major challenge for segmentation tasks. Generative models are suitable for diversifying limited datasets with new samples. In particular, to address the issue of overlapping pixel intensities of regions of interest (ROI) with other tissue types in brain MRI sequences which can make challenging the automatic pixelwise segmentation, Hamghalam et al. [56] proposed the enhancement and segmentation GAN (Enh-Seg-GAN) with the aim to generate enhanced patches, with no substantial class overlap (Fig. 9 c). The synthetically enhanced patches were derived from adaptive recalibration, encoder–decoder block (i.e., generator), and then identified from a Markovian discriminator.

Qi et al. [57] highlight and address the limitations of generating brain MRI images with tumor characteristics in previous studies [44, 46]. Specifically, the quality of the generated tumor masks is low and the actual position of the tumor compared to its mask has to be redefined manually. This can lead to changes in the image prior leading to an increase of false positives per slice. Moreover, the adversarial loss all alone is not adequate to synthesize realistic tumor images from normal MRI images [44]. Driven by the success of cycleGAN, SAG-GAN [57] was introduced to overcome these drawbacks. Its architecture includes two generators, each of which maps: (a) normal images to tumor images; (b) tumor images to normal images. The authors incorporated the idea of a semi-supervised attention mechanism into the generative network. Specifically, adding attention in the channel module allows the model to focus on channels with informative features and suppress the less useful information. Furthermore, in the architecture of the generators, an attention network aims to select the area to generate tumor and to locate the place with the tumor, leading to the generation of the probability map. On the other hand, an attention mechanism is also included in the discriminators to emphasize only the regions inside the attention map.

Guo et al. [58] proposed a SAMR framework to synthesize meaningful high-quality sequences of anatomic and molecular MRI images from arbitrary manipulated lesion information. The generator is comprised of four components; (a) a down-sampling module where the lesion segmentation maps (i.e., background, norm1al brain, edema, cavity caused by surgery and tumor) are given as input to get a latent feature map; (b) an atlas encoder that takes the analogous multi-model atlas of size 256 x 256 x 15 to get another latent feature map; (c) a set of residual blocks where the concatenation of the two latent maps is given as input to learn better transformation functions and representations; and (d) the stretch-out up-sampling module where the synthesis of MRI slices of size 256 x 256 x 5, takes place. In the other part of the adversarial learning, multi-scale PatchGAN discriminators were adopted. An expert neuroradiologist verified the pathological information of the synthesized images. Additionally, quantitative results on the external datasets (i.e., BraTS 2018) showed the superiority of the proposed method compared to other architectures [35, 46, 59]. MRI synthesis is a challenging task since radiographic features vary and pathological information includes high-frequency components. Thus, special attention is required to deal with the uncertainty [60]. To achieve this, the authors extended their previous work [58] and proposed the Confidence-Guided SAMR (CG-SAMR) [60] incorporating two crucial modules. In particular, the generator comprises of two components, an encoder and decoder with stretch-out up-sampling block. The latter component includes a synthesis module and a confidence map module. The rationale behind this is, instead of directly synthesizing MRI images from input, to initially estimate the intermediate synthesis results at half scale size, and simultaneously the corresponding confidence map is calculated by the loss function. This gives attention to uncertain regions and prevents the propagation of incorrect estimation, and therefore the synthesis of the final output is created. The discriminator components (i.e., multi-scale labelwise discriminators) remained the same as in their primary work. In addition, the proposed architecture is extended to be trained in an unsupervised fashion without the necessity of paired data (UCG-SAMR). Quantitative results reported an improvement compared with the previous method [58] and other existing architectures [35, 59]. Likewise, UCG-SAMR outperformed on pixel accuracy, SSIM and PSNR metrics; however, the network achieved the second-best performance in terms of dice score against other models [61–63].

Isocitrate dehydrogenase 1 (IDH1) mutation information is crucial for diagnosis, prognosis and guidance in clinical decisions due to observation that IDH1 mutated gliomas have an improved overall survival rate rather than with IDH1 wild type [64, 65]. However, this is molecular-level information which makes the identification a challenging task for machine learning methods. Ge et al. [66] proposed a workflow consisting of three modules to improve glioma subtype classification. The Pairwise GAN which is essentially a bi-directional cross-modality model was trained to augment data across multiple domains. Two use cases were conducted to analyze the usage of synthetic data. In the first one, the original dataset was enlarged with synthetic and in all four modalities and an increment of 2.94% and 12.73% in classification accuracy and sensitivity, respectively, was reported. However, a 1.74% decrease in specificity was reported indicating a slightly more increase of false positives. In the second, the training dataset was augmented both with synthetic images and missing scans from various modalities and an improvement on the aforementioned metrics was observed except specificity that remained same as in the baseline method. Most notably, the overall performance of the downstream task has been significantly improved by the inclusion of lesion masks in the proposed analysis.

A large number of examinations in similar datasets are not annotated on a pixel-basis, which makes training supervised DL models difficult. In [67] the authors proposed an extension to their previous adversarial architecture [66] where initially a multi-stream 2D CNN is trained to extract features from a sparsely labeled dataset followed by a graph-based semi-supervised model to assign labels to unlabeled examinations. Thus, data from unlabeled and labeled sets are fed into this pairwise GAN to enrich the original datasets. Then, during the testing phase, GAN-based data along with real labeled and unlabeled were used by multi-stream 2D CNN to learn gliomas-related imaging features. Finally, a higher-level classification module was integrated into the adversarial architecture to predict the tumor molecular subtypes.

Carver et al. [68] proposed a methodology for increasing tumor variability in terms of size, shape and location in synthetic multi-parametric MRI images, in addition to investigating how different subsets of generated images could affect segmentation performance. This architecture was trained in a supervised manner since the generator requires pixelwise annotations which in turn were derived from real MRI images. Initially, the first discriminator, a pre-trained VGG-19, was used to calculate the perceptual and per-pixel loss, whereas a second discriminator (PatchGAN) was utilized to penalize on an image patches-basis. The authors performed also qualitative analysis to further examine both the overall and inter-modality quality of synthetic images. An expert physician assessed the generated images through the Visual Turing Test and performed an in-depth analysis of pairs of synthetic and real images. It was noted that synthetic images displayed high quality with plainly defined structural boundaries; however, they were lacking in presenting the details related to edema. Nevertheless, the overall segmentation performance increased by 4.8% on the average dice similarity coefficient (DSC).

Mok et al. [69] proposed a coarse-to-fine boundary-aware generative adversarial network (CB-GAN) to synthesize high-resolution multimodal MRI images of high-grade and low-grade glioma patients. In particular, this architecture consists of two generators and four discriminators. Primarily, instead of feeding as input to the network a noise vector from a normal distribution, the authors replaced it with a semantic segmentation mask as a condition variable for introducing diversity to the generated images with different tumor shapes and preventing mode collapse. Consequently, the coarse generator is bounded to generate the primordial shape and texture of synthetic images, while a multi-task generator aims to preserve tumor boundaries by incorporating a desired invariance and robustness to the network. Multiple discriminators with different scales of input were adopted to capture both global and local information. The proposed pipeline improved the performance over traditional data augmentation methods on average by 3.5% regarding dice score and furthermore outperformed other state-of-the-art methods [70, 71] for the enhancing tumor task in terms of dice precision.

Dikici et al. [72] proposed the constrained generative adversarial network ensembles (cGANe), which is essentially an aggregate of DCGANs. The selection of which DCGAN will pass into cGANe framework was based on FD [73] computed score which had to be lower than a predefined threshold value $\omega$. The population of cGANe was determined by a brain metastasis detection algorithm [74], which was evaluated by calculating the average number of false detections per patient. To eliminate the generation of a synthetic data sample that resembles an original data sample from the training set which is crucial in terms of anonymity, the mutual information metric was used. T-SNE cluster representation visualization revealed that cGANe40 generated convincing synthetic images indistinguishable from real samples.

Kamli et al. [75] incorporated in the proposed adversarial pipeline an anonymizing model [46], to increase prediction performance in patients with glioblastoma multiforme tumor growth. The authors did not modify the tumor size and shape as in [46], because any modifications on these parameters could affect the accurate prediction of tumor growth. The Synthetic Medical Image Generator (SMIG) was trained to generate tumors in varying locations such as healthy brain regions. The authors experimentally showed that the augmentation of the dataset by up-to 80% of the samples being synthetic and 20% real data improved the segmentation performance. For this purpose, a fully automatic brain Tumor Growth Predictor (TGP) model which is based on a convolutional auto-encoder model [76] was integrated into the pipeline. Furthermore, it should be noted that pre-processing steps had a positive effect on the quantitative metrics.

Pseudoprogression (PsP) and true tumor progression (TTP) in glioblastoma multiforme (GBM) can occur after standard treatment. The distinction between them is mainly based on MRI analysis of the lesion area, which is a time-consuming procedure for clinicians. Li et al. [77] merged DCGAN and AlexNet and proposed DC-AL GAN that trained in an adversarial way on longitudinal diffusion tensor imaging (DTI) data to discriminate PsP and TTP in MRI images. In particular, the generator aims to create fake pair samples of 512 by 512 pixels in size that are similar to original data, while a modified AlexNet is placed in the role of the discriminator to extract high-level refined features. The proposed discriminator incorporates a multi-feature selection module to concatenate deep coarse features with shallow fine features. Finally, the aforementioned features were flattened and employed as input to an SVM classifier.

The number of patients, lesion types, modalities, pre-processing and downstream tasks are demonstrated in Table 1. The generative methodologies, including the deep generative architectures, and hyper-parameters are shown in details in Table 2. An evaluation comparison among the examined studies is presented in Table 3.

**Table 1:** Details of the datasets and data processing methods used in the examined studies

Author	Dataset	Patients	Lesion	Modality	Pre-processing	Objective
Beers [38]	BraTS’17	75 LGG 210 HGG	edema non-enhancing/necrosis contrast enhancing	T1w, T1ce T2w, FLAIR	isotropic resample zero mean unit variance	Synthesize
Han [39]	BraTS’16	220 HGG	tumor	T1w, T1ce T2w, FLAIR	skull striping isotropic resample resize	Synthesize
Han [41]	BraTS’16	220 HGG	tumor	T1ce	zero padding	Classification
Han [42]	BraTS’16	220 HGG	tumor	T1ce	zero padding	Classification
Han [44]	Private	180 tumor 193 normal	tumor (brain metastases)	T1ce	skull-striping crop, resize	Detection
Shin [46]	ADNI/ BraTS’15	54 LGG 220 HGG	edema non-enhancing/necrosis contrast enhancing	T1w, T1ce T2w, FLAIR	Skull-striping/ crop axially, resize	Segmentation
Chang [47]	BraTS’18	210	edema non-enhancing/necrosis contrast enhancing	T2w	-	Segmentation
Chang [48]	BraTS’18	210	edema non-enhancing/necrosis contrast enhancing	T1w, T1ce T2w, FLAIR	align isotropic resample resize	Segmentation Slice imputation
Deepak [49]	Figshare database	233	meningioma glioma pituitary tumor	T1ce	Resize	Classification
Qasim [50]	BraTS’19	335	edema non-enhancing/necrosis contrast enhancing	T1w, T2w, FLAIR	-	Segmentation
Kwon [52]	BraTS’18	210	edema non-enhancing/necrosis contrast enhancing	T2w, FLAIR	Resize	Synthesize
Chen [54]	BraTS’13/ IXI	-	edema non-enhancing/necrosis contrast enhancing/ Healthy	- T1w	-	Classification
Pesteie [55]	BraTS’17	-	edema non-enhancing/necrosis contrast enhancing	FLAIR	-	Segmentation
Hamghalam [56]	BraTS’13	30	edema non-enhancing/necrosis contrast enhancing	T1ce	-	Segmentation
Qi [57]	BraTS’19 BraTSs UNS	322 - -	tumor	T1w, T1ce T2w, FLAIR	-	Classification
Guo [58]	Private	90	GBM	T1w, Gd-T1w, T2w FLAIR, APTw	co-registration, skull-stripping N4-bias field correction MRI intensity scale standardization 2D slice extraction	Segmentation
Guo [60]	Private	100	GBM	T1w, Gd-T1w, T2w FLAIR, APTw	co-registration, skull-stripping N4-bias field correction MRI standardization	Segmentation
Ge [66]	TCGA-GBM TCGA-LGG	167	IDH genotype tumor	T1w, T1ce T2w, FLAIR	-	Classification Slice imputation
Ge [67]	TCGA-GBM TCGA-LGG/ BraTS’17	167/ 285	IDH genotype/ edema non-enhancing/necrosis contrast enhancing	T1w, T1ce T2w, FLAIR	-	Classification
Carver [68]	BraTS’18	210	GBM	T1w, T1ce T2w, FLAIR	resample, skull-stripping normalization, padding crop slices	Segmentation
Mok [69]	BraTS’15	220 HGG 54 LGG	edema non-enhancing/necrosis contrast enhancing	T1w, T1ce T2w, FLAIR	skull-stripping co-registration	Segmentation
Dikici [72]	Private	158	tumor (brain metastases)	T1ce	isotropic resample normalization	Detection
Kamli [75]	TCIA ADNI	20	GBM	T1 pre & T1 post contrast T2w, FLAIR	skull-striping normalization/standardisation registration, denoising	Prediction
Li [77]	Private	23 with PSP 61 with TTP	GBM	DTI	-	Classification

LGG, low-grade gliomas; HGG, high-grade gliomas; T1ce, T1 contrast-enhanced; FLAIR fluid-attenuated inversion recovery; T1adjacent w, T1-weighted; T2w, T2-weighted; GBM, glioblastoma multiforme; Gd-T1w, gadolinium-enhanced; APTw, amide proton transfer weighted; IDH, isocitrate dehydrogenase; PSP, pseudoprogression; TTP, true tumor progression; DTI, diffusion tensor imaging, symbol ’-’ represents that the corresponding information was not provided in the publication

**Table 2:** Details in generative methodology as presented in studies for brain tumors

Author	Architecture	Training dataset	Input	G. Arch	D. Arch	Loss function	Optimizer	Batch size	Output
Beers [38]	PGGAN	-	128-d Noise vector	nearest neighbor interpolation	CNN	W-distance	Adam	16	256 x 256
Han [39]	DCGAN/ WGAN	61,600 slices each sequence	Noise vector	Transposed CNN	CNN	GAN loss/ W-distance	Adam/ RMS-prop	64	64 x 64 128 x 128
Han [41]	PGGAN	5,036 tumor 3,853 non-tumor	Noise vector	nearest neighbor interpolation	CNN	W-distance & GP	Adam	16	256 x 256
Han [42]	PGGAN/ MUNIT/ SimGAN	154 patients 4,679 tumor & 3,750 non-tumor images	512-d Noise vector/ Synthetic images	nearest neighbor interpolation/ Style encoder & Decoder/ Refiner	CNN/ multi-scale CNN/ CNN	W-distance & GP/ MUNIT loss/ GAN loss with Self-regularization	Adam/ Adam/ SGD	16/ 1/ 10	256 x 256
Han [44]	CPGGAN	2,813 tumor images 5,963 bounding boxes 16,962 normal images	Noise vector & bounding boxes	nearest neighbor interpolation	CNN	W-distance & GP	Adam	4	256 x 256
Shin [46]	pix2pix	3,416 pairs of MRI sequences	Annotated masks	U-Net	PatchGAN	pix2pix loss	-	-	128 x 128 x 54
Chang [47]	AsynDGAN	170 patients 11,057 images	Tumor mask	9-block ResNet	PatchGAN	Custom	Adam	3	256 x 256
Chang [48]	AsynDGAN	170 patients 11,349 images	Tumor mask	9-block ResNet	PatchGAN	Custom	Adam	10	256 x 256
Deepak [49]	MSG-GAN	-	512-d Noise vector	Up-sampling & Convolutions	CNN	W-distance & GP	RMS-prop	20	128 x 128
Qasim [50]	Red-GAN	14,850 slices	Label masks	7 SPADE ResNet	PatchGAN	Hinge loss & Feature matching loss	Adam	-	256 x 256
Kwon [52]	3D-GAN	210 subjects	1000-d Noise vector	Resize-convolution / 3D-CNN	3D-CNN/ FC	Custom	Adam	4	64 x 64 x 64
Chen [54]	HybridGAN	600 slices	Fake patches & Constrained noise vector	Encoder–Decoder	CNN	Custom	Adam	30	256 x 256
Pesteie [55]	ICVAE	3,000	Real images	Encoder–Decoder	-	KLD	SGD	100	128 x 128
Hamghalam [56]	Enh-Seg-GAN	200k patches	32 x 32 patches	Recalibration block Encoder–Decoder	Markovian CNN	Custom	-	-	32 x 32
Qi [57]	SAG-GAN	225 patients	Real data	ResNet	PatchGAN	Custom	-	-	240 x 240
Guo [58]	SAMR	72 patients 1,080 instances	Lesion mask	Two encoders Residual blocks & Decoder	Six multi-scale Labelwise PatchGANs	Custom	Adam	8	256 x 256 x 5
Guo [60]	UCG-SAMR	72 patients 1,080 instances	Lesion mask & Atlases	Encoder–Decoder	Six multi-scale Labelwise PatchGANs	Custom	Adam	8	256 x 256 x 5
Ge [66]	Pairwise GAN	Mutation: 330/modality Wild-type: 672/modality	Labeled data & tumor mask	U-Net	Markovian CNN	Custom	Adam	-	128 x 128 x 4
Ge [67]	Pairwise GAN	Mutation: 33 Wild-type: 66/ HGG: 126 LGG: 45	Labeled & Unlabeled data tumor mask	U-Net	Markovian CNN	Custom	Adagrad	9	128 x 128 x 4
Carver [68]	GAN model	164 patients	Semantic labels & Real images	U-Net	VGG-19 & PatchGAN	Custom	-	-	256 x 256
Mok [69]	CB-GAN	220 HGG 54 LGG	Semantic labels	Convolution Residual block Transposed Convolution	CNN	Custom	Adam	-	-
Dikici [72]	cGANe	-	Noise vector	Transposed CNN	CNN	BCE	Adam	8	16 x 16 x 16
Kamli [75]	SMIG	17 patients/ 3,416 pairs	Lesion tumor volume / Normal volume	U-Net	PatchGAN	-	-	-	256 x 256 x 4
Li [77]	DC-AL GAN	-	100-d Noise vector	Transposed CNN	AlexNet	Custom	Adam	64	512 x 512

G. Arch, Generator Architecture; D. Arch, Discriminator Architecture; 128-d, 128-dimensional; PGGAN, Progressive Growing of Generative Adversarial Networks; CNN, Convolutional Neural Network; W-distance, Wasserstein distance, Adam, Adaptive Moment Estimation; DCGAN, Deep Convolutional GAN; WGAN, Wasserstein GAN; RMS-prop, Root-Mean-Squared propagation; GP, Gradient Penalty; MUNIT, Multimodal Unsupervised Image-to-Image Translation; SimGAN, Simulated and unsupervised images through adversarial training, SGD, Stochastic Gradient Descent; CPGGAN, Conditional Progressive Growing of GAN; AsynDGAN, Asynchronized Discriminator GAN; ResNet, Residual Network; SAG-GAN, Semi-supervised Attention-Guided GAN; SAMR, Synthesis of Anatomic and Molecular MRI images network; UCG-SAMR, Unsupervised Confidence-Guided SAMR; AdaGrad, Adaptive Gradient algorithm; MSM-GAN, Multi-Scale gradient GAN; RMS-prop, Root-Mean-Squared propagation; Enh-Seg-GAN, Enhancement and Segmentation GAN; VGG, Visual Geometry Group; CB-GAN, Coarse-to-fine Boundary aware GAN; cGANe constrained GAN ensembles; SMIG, Synthetic Medical Image Generator; DC-AL GAN, Deep Convolutional AlexNet GAN; FC, Fully Connected; ICVAE, Independent Conditional Variational Auto-Encoder; BCE, Binary Cross-Entropy; KLD, Kullback–Leibler Divergence, symbol ’-’ represents that the corresponding information was not provided in the publication

**Table 3:** Evaluation performance of generative methods presented in the examined studies for brain tumors

Author	Quantitative metrics			Qualitative metrics	Downstream task
	Direct for SD	Indirect prior to SDA	Indirect after SDA	Experts/statistics(%)	Model
Beers [38]	N/A	N/A	N/A	Plotted samples	N/A
Han [39]	N/A	N/A	N/A	Physician/53.0	N/A
Han [41]	N/A	Acc: 0.900 Sen: 0.852 Spe: 0.970	Acc: 0.910 Sen: 0.866 Spe: 0.976 (& classical DA)	Physician/78.5	ResNet-50
Han [42]	N/A	Acc: 0.931 Sen: 0.909 Spe: 0.958	Acc: 0.948, Sen: 0.936, Spe: 0.984 (GAN-based DA) Acc: 0.967, Sen: 0.974, Spe: 0.988 (GAN-based & classic DA)	Physician/76.0 t-SNE	ResNet-50
Han [44]	N/A	Sen: 0.67	Sen: 0.77	Three Physicians/ Ph1: 91.0, Ph2: 96.0, Ph3: 100 t-SNE	YOLOv3
Shin [46]	N/A	Dice: 0.64 ± 0.14	Dice: 0.80 ± 0.07	Plotted samples	pix2pix
Chang [47]	N/A	Dice: 0.748 Sen: 0.798 Spe: 0.995 HD(95): 12.85	Dice: 0.704 Sen: 0.729 Spe: 0.995 HD(95): 14.94	Plotted samples	U-Net
Chang [48]	N/A	Dice: 0.808 Sen: 0.785 Spe: 0.996 HD(95): 11.95	Dice: 0.773 Sen: 0.742 Spe: 0.996 HD(95): 16.44	Plotted samples	U-Net
Deepak [49]	N/A	Balanced Acc: 0.903	Balanced Acc: 0.931	Plotted samples	CNN
Qasim [50]	N/A	N/A	Dice: 0.779	Plotted samples	U-Net
Kwon [52]	MMD: 0.072 MS-SSIM: 0.843	N/A	N/A	PCA	N/A
Chen [54]	IS: 2.32 ± 0.04 FID: 139 ± 4.5 KID 0.144 ± 0.006	Acc: 0.901	Acc: 0.888	Plotted samples	CNN
Pesteie [55]	N/A	Dice: 0.80 ± 0.33 Hausdorff: 2.78 ± 3.24	Dice: 0.88 ± 0.26 Hausdorff: 2.16 ± 2.6	Plotted samples	U-Net
Hamghalam [56]	SSIM: 0.7245 PSNR: 22.23	N/A	Dice: up-to 0.89 Sen: up-to 0.96 PPV: up to 0.83	Plotted samples	Pixelwise Classifier
Qi [57]	N/A	Acc: up-to 0.933 AUC: up-to 0.961	Acc: up-to 0.950 AUC: up-to 0.969	Plotted samples	ResNet-18
Guo [58]	N/A	Dice: up-to 0.673 HD(95): as low as 7.078 Sen: up-to 0.678 Spe: 0.999	Dice: up to 0.821 HD(95): as low as 1.568 Sen: up-to 0.807 Spe: 0.999	Neuroradiologist/72.1	U-Net
Guo [60]	Pixel Acc: up-to 0.774 SSIM: 0.812 PNSR: 21.8	Dice: up-to 0.672	Dice: up-to 0.840	Plotted samples	U-Net
Ge [66]	PSNR: up-to 26.14 DAEF: as low as 132.40	Acc: 0.852 ± 0.322 Sen: 0.690 ± 0.137 Spe: 0.939 ± 0.389	Acc: 0.888 ± 0.637 Sen: 0.818 ± 0.111 Spe: 0.921 ± 0.477	Radiologist/-	Multi-stream 2D CNN
Ge [67]	N/A	Acc: 0.853 ± 0.443, Sen: 0.735 ± 0.927 Spe: 0.909 ± 0.525 Acc: 0.895 ± 0.142, Sen: 0.782 ± 0.435 Spe: 0.936 ± 0.275	Acc: 0.865 ± 0.424, Sen: 0.737 ± 0.815 Spe: 0.927 ± 0.345 Acc: 0.907 ± 0.142, Sen: 0.843 ± 0.659 Spe: 0.930 ± 0.142	Plotted samples	Multi-stream 2D CNN
Carver [68]	MSE: as low as 18.4 MAE: as low as 22.8 SSIM: up-to 0.794 PSNR: 43.1	N/A for Dice Sen: up-to 0.89, Spec: up-to 0.99	Dice: increase of 0.48 Sen: up-to 0.90, Spec: up-to 0.99	Physician/26.3	U-Net
Mok [69]	N/A	Dice: 0.79	Dice: 0.84 (GAN-Based DA) Dice: 0.81 (classical DA)	Plotted samples	U-Net
Dikici [72]	FD > 0.4	AFP: 9.12	AFP: 9.53	t-SNE	BM-detection framework
Kamli [75]	N/A	Recall: 0.643 Precision: 0.625 Dice: 0.641	Recall: 0.699 Precision: 0.717 Dice: 0.723	Plotted samples	TGP
Li [77]	N/A	N/A	Acc: 0.920 AUC: 0.947	t-SNE	SVM

SD, Synthetic Data; SDA, Synthetic Data Augmentation; Acc, Accuracy; Sen, Sensitivity; Spe, Specificity; t-SNE, t-distributed Stochastic Neighbor Embedding; HD(95), Hausdorff Distance; AUC, Area Under the Curve; SSIM, Structural Similarity Index Measure; PSNR, Peak Signal-to-Noise Ratio; DAEF, Distance to the real images based on Autoencoder Features; PPV, Predicted Positive Value; MSE, Mean Square Error; MAE, Mean Absolute Error; FD, Frechet Distance; AFP, Average False Positive; MMD, Maximum Mean Discrepancy; MS-SSIM, Multi-Scale Structural Similarity Metric; PCA, Principal Component Analysis; IS, Inception Score; FID, Frechet Inception Distance; KID, Kernel Inception Distance; N/A, Not applicable; Symbol ’-’ represents that the corresponding information was not provided in the publication

### Prostate

DCGANs and cGANs are novel variants of generative models published after their first appearance [22]. Kitchen et al. [78] employed DCGANs in 2017 to synthesize 16 by 16 prostate MRI patches across three modalities such as T2, ADC, $K^{trans}$. One year later Hu et al. [79] employed cGANs that take Gleason scores as a condition in the training process with the aim to synthesize focal prostate diffusion images of size 32 by 32.

ADC values derived from diffusion-weighted MRI are useful non-invasive biomarkers for accurately assessing the clinical significance (CS) of suspicious glands for prostate cancer (PCa) [80]. However, such data are often scarce and thus it is limiting the usage of DL architectures. Wang et al. [81] present a stitch AD-GAN to synthesize the ADC data at a size of 64 by 64. Initially, the target image space was divided into subspaces each of which had a lower scale in order to reduce the complexity of the data manifold. Thus, instead of directly synthesizing the image in the target space that would affect the quality of data, it is generated four 32 by 32 sub-images in the divided subspaces. Then, a nonparametric Stitch layer was employed to interlace these sub-images into the full-size target image. Additionally, the discriminative module consists of two critic networks: (a) the first minimize Wasserstein distance between synthetic and real CS PCa data; and (b) the second maximize the auxiliary distance JSD among synthetic CS PCa and non-CS PCa. By incorporating a StitchLayer and the aforementioned loss functions the network was capable of synthesizing full images with global structure and precise local information.

Yang et al. [82] presented a novel bi-parametric (i.e., ADC-T2w) image generation network based on semi-supervised learning. In particular, the proposed framework consists of two generative modules that synthesize corresponding images in the two modalities in sequential order. The sequential synthesis mainly consists of three modules (a) a pre-trained on ImageNet [2] Inception-V3 network that extracts hierarchical features of both real and fake images to measure the complexity of two modalities; the modality with the lower complexity is synthesized first (b) an encoder which maps a real image of each modality to a low-dimensional latent vector and (c) a synthesizer which first decodes the latent vector to a fake image and then translates it to another modality. Semi-supervised sequential synthesis of bi-modality images achieved superior performance in all evaluation metrics and classification accuracy when compared with supervised sequential GANs, unsupervised sequential GANs and parallel GANs. Wang et al. [83] combined these studies [81, 82] and proposed an improved semi-supervised architecture to synthesize mp-MRI data with sufficient diversity to include meaningful CS PCa from a small amount of training data. Initially, a decoder derives low-dimensional ADC maps from 128-d latent vectors, then a StitchLayer converts the low-dimensional ADC maps to a full-size ADC image, and finally, a U-Net is used as an image translator to convert ADC image to a paired T2w. Complexity measurer was excluded as ADC maps are proven to be easier to synthesize first due to low spatial resolution.

Fernandez-Quilez et al. [84] proposed a semiautomatic pipeline with two generative models in a sequential fashion to generate synthetic pairs of T2-weighted prostate MRI and their corresponding whole gland mask. Initially, a DCGAN model was trained on PROMISE 12 dataset to synthesize whole prostate gland masks. The selection criteria for synthetic masks are based on the visual appearance and done manually. Afterward, a pix2pix architecture converted the synthetic whole gland masks into T2-weighted modality leading up to 10 thousand synthetic paired data. For evaluation a segmentation task was performed, where a U-Net architecture trained with multiple data including real data, synthetic, classical augmentation and combinations of them.

Taking into consideration the DCGANs drawbacks, Yu et al. [85] proposed CapGAN which features two major modifications. First, the capsule network replaced the CNN as a discriminator to better achieve an equivariant representation of images that is robust to the changes in the pose and spatial relationship of objects in the images. Second, the least-squares loss was adopted for both generator and discriminator to address the vanishing gradient problem of the sigmoid cross-entropy loss function and simultaneously generate higher-quality images. Furthermore, to evaluate the synthetic images both qualitative and quantitative metrics were introduced. Qualitative evaluation was conducted through visual inspection by two experts, while quantitative evaluation was performed in terms of KL divergence by calculating the probability distribution of synthetic and real images based on a pre-trained SVM classifier. The downstream task such as classification was applied via a combination of LENet [86] and NIN [87] architectures. The whole framework was applied both to prostate and simulated brain MRI images.

To address the cross-client variation problem in medical image data, Yan et al. [88] proposed a variation-aware federated learning (VALF) framework. Three different GAN architectures were employed to address privacy concerns. First, the data complexity of all clients was calculated to define the target image space. In detail, a WGAN with gradient penalty was trained to generate synthetic data and PCA with t-SNE was applied to extract discriminative imaging features. The complexity score for each client was defined by calculating L2 distance between the features of generated and original data. The client with the lowest complexity was selected as the target image space. Afterward, to share the defined image space with other clients, a collection of images is synthesized via a privacy-preserving-adversarial network (PPWGAN-GP), and a subset of them which can effectively capture the characteristics of the raw images but without leaking the privacy is selected. Finally, by a modified cycle-GAN, the corresponding raw images of each client are converted into target image space defined by the shared synthetic images.

### Pancreas

Gao et al. [89] employed a GAN in a recent study [32] to improve a pancreatic neuroendocrine tumor (pNET) differentiation model. The generator incorporated a fully connected layer followed by several fractionally strided convolutional layers with a kernel of 4 by 4 pixels. The input vector of 100 noise values was selected from a normal distribution to be transformed by the generator into synthetic T1ce patches of 56 by 56 pixels. The authors emphasize the value of the generated images, which can assist with the difficult task of gathering radiological examinations of rare diseases. The proposed analysis, which incorporates a generative model and deep learning classification, demonstrated the capacity to discriminate among the World Health Organization (WHO) grades of pNET on T1 contrast-enhanced MRI. The generated patches were evaluated by two radiologists to ensure the quality of the training samples. In another work, [90] a DCGAN was used to increase the extracted ten thousand patches of regions of interest up to twenty-five thousand training samples for pancreatic disease classification. The original dataset was comprised of 448 patients with T1-weighted dynamic contrast-enhanced MRI examinations and an external validation set of 56 patients. A total of twenty-three diseases were grouped into seven classes. Since the largest out of seven groups of patches was the carcinomas, it was left out of the generation process to improve the imbalanced dataset. Consequently, six generative models were trained on the remaining groups, independently for each class. This resulted in a balanced training set across all seven groups. One radiologist examined the generated images to ensure the validity of the process.

### Breast

Generative models have been implemented for synthesizing multi-parametric breast MRI image patches by Haarburger et al. [91]. Two custom GAN architectures were employed to generate sequences of T2 and T1-DCE image patches that were conditioned on healthy tissue, benign and malignant lesions. Initially, an experienced radiologist segmented manually all suspicious and non-lesion areas on every slice, leading to a dataset of 401,525 patches in total. DCGAN and WGAN were trained to synthesize patches 64 by 64. The generated samples were evaluated qualitatively by expert clinicians as well as quantitatively. Despite most of the patches being morphologically realistic, it was also observed that synthesized images included fat-shift artifacts. In terms of quantitative metrics, DCGANs were superior to WGANs, but qualitatively minor differences were observed.

### Liver

Sun et al. [92] implemented a manifold matching generative adversarial network (MM-GAN) for augmenting the training set up to 500%. The effect of different levels of synthetic data ranging from 0 to 500% was also tested in segmentation tasks on two datasets: BRATS17 and LIVER100. In particular, the performance in terms of DSC of the glioma segmentation (T1ce) was improved for the whole tumor by 0.17 and the tumor core by 0.16 on the unseen testing set. Additionally, only a small fraction of the original dataset (29 samples) was used for fine-tuning the model that was trained exclusively on synthetic data without compromising the segmentation performance. The synthetic data were assessed visually by observing the brain structure and the retained details (namely the cerebrum, cerebellum, diencephalon, brainstem, sulci, gyri). A key aspect of the synthetic images was that the brain anatomy was adapted with respect to the given segmentation mask while still displaying a noticeable difference in appearance between real MRI images.

Details about the examined datasets, pre-processing methods, deep generative model architectures, and a comparative analysis are shown in Tables 4, 5 and 6.

**Table 4:** Details of the datasets and data processing methods used in the examined studies

Authors	Dataset	Patients	Anatomical area	Lesion	Modality	Pre-processing	Objective
Kitchen [78]	SPIE PROSTATEx challenge 2016	-	Prostate	-	ADC, T2, $K^{trans}$	Normalization [0,1]	Synthesis
Hu [79]	Private	104	Prostate	Gleason Scores (0 to 9)	Diffusion images	Rotation Normalization Flipping	Synthesis
Wang [81]	Private / PROSTATEx	CS PCs: 134 non-CS PCa: 226	Prostate	CS & BPH	ADC	-	Classification
Yang [82]	Private / PROSTATEx	CS PCs: 134 non-CS PCa: 226	Prostate	CS & BPH	ADC, T2w	Non-rigid registration to ADC & T2w, manually crop ROI	Classification
Wang [83]	TJPCa / PROSTATEx	CS PCs: 134 non-CS PCa: 226	Prostate	CS & BPH	ADC, T2w	Crop & align the ROI, Resize pairs	Classification Localization
F-Quilez [84]	PROMISE 12	80	Prostate	Whole gland	T2w	Linear interpolation Outlier removal Normalize [0,1] CLAHE	Segmentation
Yu [85]	PROSTATEx’17	-	Prostate	Benign & malignant	T2w, ADC, DCE	Crop the ROI	Classification
Yan [88]	LocalPCa/ PROSTATEx	135/ CS PCa: 64 non-CS PCa: 124	Prostate	CS PCa non-CS PCa	ADC	-	Privacy Preservation & Classification
Gao [89]	Private	96 pNET	Pancreas	Neuroendocrine Tumors	T1ce	-	Classification
Gao [90]	Private	Train set: 398 Internal val set: 50 External val: 56	Pancreas	7 disease Groups	T1ce	-	Classification
Haarburger [91]	Private	408	Breast	Benign Malignant Healthy	T1w, T1ce, T2w	Rescale intensities Resample resolution Crop to patches	Synthesize
Sun [92]	Liver100/ BRATS17	285/ 100	Liver/ Brain	7 groups of lesions (23)/ HGG/LGG	T1w,T1ce, T2w, & FLAIR	Normalize to zero mean & unit variance Crop	Segmentation

ADC, Apparent Diffusion Coefficient; CS, Clinically Significant; PCa, Prostate Cancer; T2w, T2-weighted; DCE, Dynamic Contrast Enhanced; BPH, Benign Prostatic Hyperplasia; ROI, Region of Interest; pNET, pancreatic Neuroendocrine Tumor; T1ce, T1 contrast-enhanced; val, validation; LGG, Low-Grade Gliomas; HGG, High-Grade Gliomas; FLAIR, fluid-attenuated inversion recovery; Symbol ’-’ represents that the corresponding information was not provided in the publication

**Table 5:** Details in generative methodology as presented in studies with anatomical regions such as prostate, liver, breast and pancreas

Authors	Architecture	Training dataset	Input	G. Arch	D. Arch	Loss function	Optimizer	Batch size	Output
Kitchen [78]	DCGAN	330 Patches	25-d Noise vector	Transposed CNN	CNN	CE	Adam	200	16 x 16 x 3
Hu [79]	ProstateGAN	1490 Diffusion images	100-d Noise vector	Transposed CNN	CNN	Conditional GAN loss	Adam	64	32 x 32
Wang [81]	StitchAD-GAN	483 CS 1942 non-CS	128-d Noise vector	Transposed CNN	Two CNNs	W-distance & JSD	-	-	64 x 64
Yang [82]	Semi-supervised Sequential GAN	483 CS	128-d Noise vector & Encodings of real data	Decoder & image translator	CNN	W-distance & L1	Adam	32	64 x 64
Wang [83]	Semi-supervised sequential GAN with StitchLayer	483 CS 1942 non-CS	128-d Noise vector & Encodings of real data	Decoder & U-Net translator	CNN	W-distance & L1 & JSD	Adam	32	64 x 64
F-Quilez [84]	DCGAN/ pix2pix	50	100-d Noise vector/ Synthetic mask	Transposed CNN/ U-Net	CNN/ PatchGAN	BCE/ pix2pix loss	Adam	32/ 1	256 x 256
Yu et [85]	CapGAN	24.000 patches each modality	100-d Noise vector	Transposed CNN	Capsule Network	LSE	Adam	-	35 x 35
Yan [88]	PPWGAN-GP	up-to 1,688	Noise vector	Transposed CNN	CNN	Custom	Adam	24	-
Gao [89]	DCGAN	G1: 547, G2: 1265 G3: 164 (patches to PNG)	100-d Noise vector	Transposed CNN	CNN	-	Adam	64	56 x 56
Gao [90]	DCGAN	10293 (patches to PNG)	100-d Noise vector	Transposed CNN	CNN	-	Adam	64	88 x 88
Haarburger [91]	DCGAN/ WGAN	401.525 patches	Noise vector	Resize-Convolution/ Transposed CNN	CNN	GAN loss/ W-distance	Adam/ RMS-prop	64	64 x 64
Sun [92]	MM-GAN	210 HGG 75 LGG	Label maps	3D U-Net	3D CNN	LSE	Adam	1	200 x 160 x 150

DCGAN; Deep Convolutional GAN; CNN; Convolutional Neural Network; CE, Cross-Entropy; Adam, Adaptive Moment Estimation; CS, Clinically Significant; W-distance, Wasserstein-distance; JSD, Jensen-Shannon Divergence; CapGAN, Capsule GAN; LSE, Least Square Error; PPWGAN-GP, Privacy Preserving-adversarial network; RMS-prop, Root-Mean-Squared propagation; Symbol ’-’ represents that the corresponding information was not provided in the publication

**Table 6:** Evaluation performance of generative methods presented for various anatomical regions in the examined studies

Author	Quantitative metrics			Qualitative metrics	Downstream task
	Direct for SD	Indirect prior to SDA	Indirect after SDA	Experts/statistics(%)	Model
kitchen [78]	N/A	N/A	N/A	Plotted samples	N/A
Hu [79]	N/A	N/A	N/A	Plotted samples	N/A
Wang [81]	N/A	Acc: 0.92 (& classical DA)	Acc: 0.95	Plotted samples	FC-ANN
Yang [82]	FID: 179.54 ± 5.38 IS: 2.61 ± 0.24 MID: 0.011 ± 0.006	N/A	Acc: 0.93 ± 0.45	Three Radiologists/ R1: 4.4 FPR R2: 91.3 FPR R3: 7.8 FPR	FC-ANN
Wang [83]	IS: 2.24 ± 0.03 FID: 178.2 ± 3.7 SCA: 0.944 ± 0.5	N/A	Acc: 0.90 Sen(0.1): 0.26 Sen(1.0): 0.80	Three radiologists/ R1: Sen 26.0 SCA 63.0 R2: Sen 24.0 SCA 61.0 R3: Sen 82.0 SCA 89.0	FC-ANN CNN detector
F-Quilez [84]	HD: 8.10	Dice: 0.678 MSD: 3.16 VDSC: 0.543	Dice: 0.737 MSD: 1.16 VDSC: 0.693	Visual evaluation	U-Net
Yu [85]	KLD: as low as 0.73	N/A	Acc: up-to 0.892 AUC: up-to 0.885	Two radiologists/(N/A)	LENet-NIN
Yan [88]	L2 Distance (no values)	N/A	Acc: up-to 0.983 AUC: up-to 0.997	t-SNE & PCA visualization	CNN
Gao [89]	N/A	N/A	AAcc: 0.8105 ma-AUC: 0.8847	Two radiologists/(N/A)	CNN
Gao [90]	N/A	N/A	IVPL AAcc: 0.715, ma-AUC: 0.9204 EVPL AAcc: 0.794, ma-AUC: 0.9451 IVPAL AAcc: 0.700, ma-AUC: 0.8250 EVPAL AAcc: 0.767, ma-AUC: 0.8646	Radiologist IV AAcc: 0.820, ma-AUC: 0.8950 EV AAcc: 0.839, ma-AUC: 0.9063	InceptionV4
Haarburger [91]	FID: as low as 20.23 1-NN: up-to 0.268	N/A	N/A	Radiologist /70.0 Layperson /76.7	N/A
Sun [92]	N/A	Dice: 0.6856 ± 0.18	Dice: 0.6903 ± 0.20	Plotted samples	3D U-Net

SD, Synthetic Data; SDA, Synthetic Data Augmentation; Acc, Accuracy; FID, Frechet Inception Distance; IS, Inception Score; MID, Mutual Information Distance; FPR, False Positive Ratio; SCA, Slice-level Classification Accuracy; Sen, Sensitivity; Spe, Specificity; HD, Hausdorff Distance; MSD, Mean Surface Distance; VDSC, mean Volumetric DSC; KLD, Kullback–Leibler Divergence; AUC, Area Under the Curve; t-SNE, t-distributed Stochastic Neighbor Embedding; PCA, Principal Component Analysis; IVPL, Internal Validation Patch Level; EVPL, External Validation Patch Level; IVPAL, Internal Validation PAtient Level; EVPAL, External Validation PAtient Level; AAcc, Average Accuracy; ma, micro-averaging; 1-NN, Nearest Neighbor; N/A, Not applicable; Symbol ’-’ represents that the corresponding information was not provided in the publication

## Discussion

**Fig. 10:**
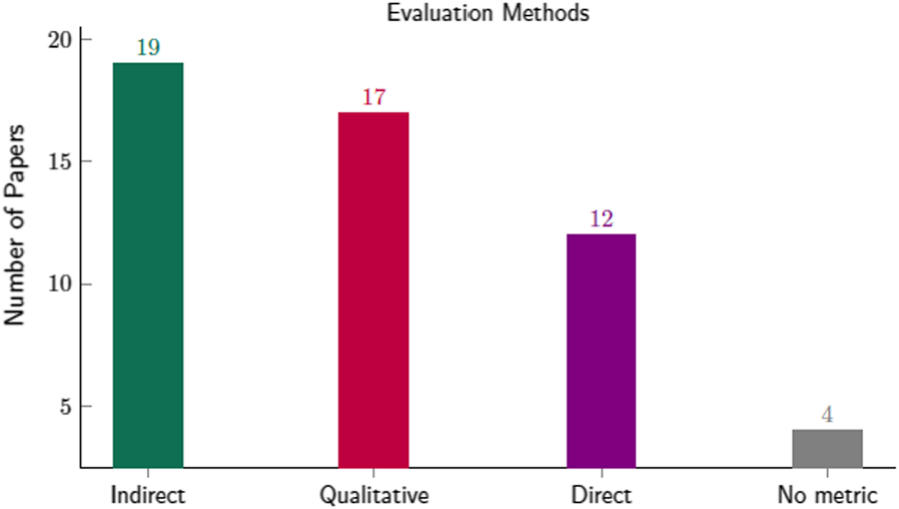
Evaluation methods for assessing the generative process: an indirect metric for the downstream task (i.e., classification, segmentation, detection, etc.) where is calculated the performance prior to and after sample generation. Qualitative analysis is where expert clinicians assess the generated images with statistical methods or the via Visual Turing Test. Direct assessment of generated samples with image quality metrics (e.g., MSE, FID, IS, etc.), and studies without any metric

Deep learning applications regarding medical image analysis applications require big databases from different acquisition protocols in order to effectively capture the intra-class variability in lesion types. This is especially relevant in oncology, where in many cases the natural prevalence of the disease, anatomical variability and tumor heterogeneity cannot be modeled due to the limited available data. Deep generative models can potentially alleviate this drawback by capturing the distribution of each lesion and synthetically augmenting the patient cohort with a robust and diverse sample distribution.

### Synthetic data evaluation

Three types of synthetic data evaluation and their combinations were followed in the examined studies: a) direct by specific image quality metrics (MSE, MAE SSIM, etc.): b) indirect by examining the delta of the performance prior and after samples generation: and c) qualitative by either expert clinicians or by plotting cluster distribution of the generated samples.

A direct assessment of the generative task using statistical metrics was performed by twelve studies [52, 54, 56, 60, 66, 68, 72, 82–85, 91] prior to downstream model training in order to verify the validity and quality of the generated samples. These types of metrics provide an objective and quantitative process of assessing the generative models, allowing comparison among different methodologies.

**Fig. 11:**
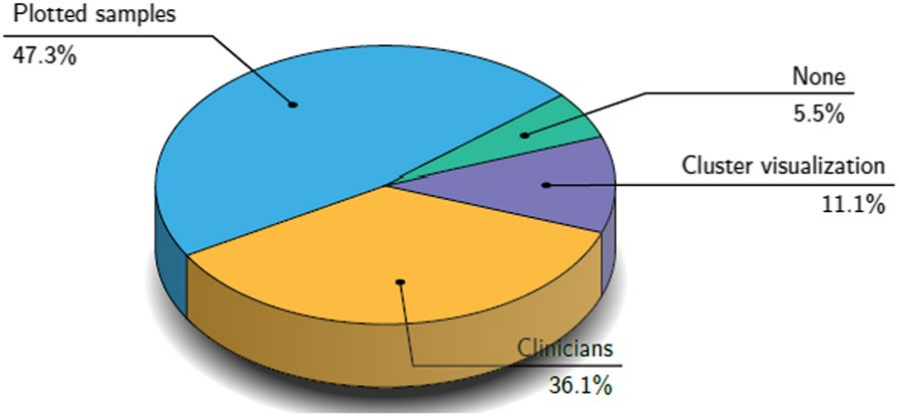
Qualitative methods were used in the examined studies for evaluating the generated samples. Almost half of the examined studies evaluated the synthetic images by visualization, whereas 36.1% employed expert clinicians to assess the generated samples using statistical methods or an operator-assisted device that produces a stochastic sequence of binary questions from a given test image (i.e., Visual Turing Test). The 11.1% used cluster visualization methods such as PCA and t-SNE, and a small percentage (5.5%) did not use any qualitative method to assess the synthetic images

On the contrary, the majority of the examined studies, as illustrated in Fig. 10, incorporate an indirect evaluation of the generated images. Additionally, seventeen [38, 46–49, 54–57, 60, 67, 69, 75, 78, 79, 81, 92] of these studies visualize a selection of the synthetic samples, and thirteen [39, 41, 42, 44, 58, 66, 68, 82, 83, 85, 89–91] were qualitatively evaluated by experienced clinicians, as illustrated in Fig. 11. However, due to the large amount of synthetic data generated, this sort of qualitative evaluation is prone to errors and inter-observer variability [90]. Furthermore, some studies that employ expert clinicians to assess the generated samples report large variability in the scores [82, 83].

A significant number (nineteen) [38, 39, 44, 46, 49, 50, 52, 54, 56, 60, 72, 77–79, 81, 89–92] of those papers did not provide sufficient statistical metrics (four of them none) [38, 39, 78, 79] for assessing the impact of synthetic data on model performance. This is likely to have led to the insufficient evaluation of the generalization status in the examined downstream tasks with reduced performance in the unseen data. Because deep learning-based techniques are known to be prone to overfitting and noisy information memorization, this is a significant disadvantage for model trustworthiness and robustness of the generative models.

### Limitations of this review

This study has some limitations. It was particularly difficult to identify studies that incorporated GANs or VAE for data augmentation since, in many papers, it was merely a component of their overall study pipeline or was only referenced briefly in a paragraph with little information. Furthermore, in the majority of studies, the authors did not provide the necessary information (analysis protocol, hyperparameters, measurements, etc.) that would allow us to fully assess the quality of each study and objectively evaluate their findings. As a result, most of these experiments are impossible to replicate. The original source code or custom datasets are not publicly available for the examined manuscripts, but only in a small number of studies, making extraction of the required hyperparameter problematic.

### Limitations of the reviewed papers

There are different strategies to mitigate the limited population of the original dataset, including subsampling of examinations at a slice level (from 3D volume to 2D slices, twenty-six studies) [38, 39, 41, 42, 44, 47–50, 54, 55, 57, 58, 60, 66–69, 75, 77, 79, 81–84, 88] and at a patch level (from tumor to sub-regions of the tumor, seven studies) [56, 72, 78, 85, 89–91]. These subsampling techniques can result in the loss of key features of tumor heterogeneity, significant voxel-based and spatial information with morphological features such as sphericity, shape and volume. This is likely to negatively impact the generalization ability of both the generative model and decision support systems.

The inherent heterogeneity of cancer images emanating from specific genetic traits, local mutational diversity, varying shape attributes, unclear boundaries, multiple subtypes and stages can significantly affect clinical outcomes. Because of all these parameters, capturing discriminative imaging markers for assessing the output variables can become challenging when generating images that include highly heterogeneous regions of interest.

Random noise vectors have been utilized as input for data generation in twenty-one [38, 39, 41, 42, 44, 49, 52, 54, 72, 77–79, 81–85, 88–91] and pixel-level lesion annotations in twelve studies [46–48, 50, 58, 60, 66–69, 75, 92]. Although this ensures the generation of different types of tissue, including tumor regions, it may lead to less variety in terms of shape and volume of the examined anatomical regions. A potential solution to this issue was proposed by Pesteie et al. [55] in which random deformation of the semantic segmentation mask was performed prior to generating new samples.

The reproducibility of deep learning models in medical imaging is a major challenge since decision support systems must adhere to the relevant legislation and be licensed by the respective regulatory bodies. Many studies, as evident by tables 1, 2 and 4, 5 , provide incomplete experimental protocol information with missing key parameters and data processing details. In addition, many studies are based on proprietary datasets, making comparisons with similar approaches challenging. Open datasets and publicly available source code repositories could, to an extent, address these issues and accelerate progress with respect to the current state-of-the-art methods. The open-source code from reviewed papers on the GitHub repository is presented in Table 7.

Class imbalance on a patient-basis regarding the examined disease can potentially result in lower performance in the minority class in both the generative model and downstream tasks [67, 90]. Consequently, this could likely compromise the diagnostic value of the deep model. Stemming from the limited patient data in most datasets, the lack of anatomical diversity is a critical issue in oncological imaging since the available tumor pixels are far less than the other types of tissues in the examined volume of interest [44].

A trade-off between signal quality and noise in the generated MRI examinations is a key element during the convergence of generative models to capture the granular imaging patterns in each class distribution. Constraints during generation might be implemented to ensure that the intensities of pixels are uniform and realistic [93]. There are also concerns regarding the image quality of the generated samples (low-resolution, distortion, blurriness, etc.) [90, 94, 95]. Additionally, variations [60] in spatial resolution and pixel coordinates among MRI examinations may compromise the generalizability of the analysis when raw data are used. Thus, resampling to harmonize spacing is a necessary pre-processing task for any (2D or 3D) convolutional deep model, but this can also substantially affect the underlying hidden tumor patterns in MRI images [90].

Deep models trained on a single medical center might capture biased distributions. Thus, external validation sets are of paramount importance for generalization [58, 89, 90]. Additionally, evaluating generated images by expert radiologists cannot be always considered a feasible option due to their limited available time, the high-dimensionality of MRI images and the subjective nature of the tasks often requiring multiple clinicians in order to minimize inter-observer variability. Additionally, as is evident in Table 6, the difference in qualitative scoring for the generated samples by expert clinicians can be substantial.

**Table 7:** Studies with available open-source code on GitHub repository

Authors	Open-source code
Yi [27]	3DUnetCNN
Shin [46]	pix2pix
Chang [47]	AsynDGAN
Guo [60]	CG-SAMR
Qasim [50]	Red-GAN
Kwon [52]	3D-GAN
Pesteie [55]	ICVAE Theano
Yang [82]	Bi-Modality-Medical-Image-Synthesis

### Advancing generative models for radiology applications

Novel quantitative and qualitative methods should be developed [96] to provide insights not only about how realistic a generated image is but also to ensure that regions of interest are anatomically correct [93] and a true representation of MRI scans. Additionally, generating 3D MRI volumes instead of 2D slices [46, 52, 92] can significantly improve the convergence of the downstream tasks since imaging features based on three-dimensional raw data can increase robustness and generalizability. Additionally, key advancements in generative models include the improvement in the fidelity [60, 77, 93, 97] and reduction of the smoothed patterns [93] of MRI images via denoising or other voxel-based techniques.

Ge et al. [67] suggest that GANs can be extended to capture rare genetic alterations that have a significant impact on assessing the response to targeted treatments. Wang et al. [81] employed a stitch layer in the generator to address the difficult-to-optimize problem in most GANs for high-dimensional image synthesis in prostate MRI. Different techniques have been introduced to improve image quality [91] and enhance deep generative model convergence [42, 49, 57–60, 68].

GANs trained on multicentric MRI data can benefit from scanner variability and further improve generalization of the targeted task [60]. Accordingly, enhancing the render process of MRI itself by utilizing the raw k-space data [98] will advance the current acquisition and reconstruction process, enabling a more optimized quantitative analysis.

Regional legislative frameworks for privacy are posing significant challenges in medical data analysis. Deep generative models can assist in providing full anonymity of medical data [44, 46–48, 72, 75, 88], even on an image level, making it easier to share them specifically the synthetic version of them.

### Research challenges and future directions

Despite the active research of generative models on MRI image analysis, non-trivial challenges still remain. Future studies should examine a more diverse patient cohort to capture the tumor variability in terms of shape, location, anatomical region, genetic background, histological subtypes and other clinically significant parameters.

In particular, 35% of the examined studies, as illustrated in Fig. 4, were either unclear about their patient stratification protocol or had a high risk of introducing selection bias like focusing only on large lesions such as high-grade glioma. Data augmentation for rare tumors in anatomical locations such as the pancreas, renal and bones needs further investigation as only a handful of studies were reported in Table 4.

The existing generative models have been developed to converge with selection criteria that are ROI size-restricted. The introduction of architectures that can capture the high variability of tumors is crucial. In particular, more effort should be invested into generating MRI examinations with small lesion ROIs such as low-grade gliomas, lung nodules and other similar-sized neoplasms. Oncology imaging is also characterized by the fact that the population, scanner manufacturers and acquisition methods at different sites vary a lot. Generative models fitted on a diverse set of data could achieve an improved and generalized representation of data distributions that are invariant to these differences.

However, the heterogeneity of data might not be preserved in the generated distribution and artifacts might be introduced due to the drawbacks of current cost functions and architectures. Thus, future architectures should be employed on tumor datasets along with new metrics that are better suited for robust evaluation of generative models, not just for image quality but also for assessing diversity in the generated dataset.

The computational cost and the required time for developing 3D generative models are high. This limits the majority of studies to 2D models and, therefore, key volumetric features cannot be captured by the synthetic distribution. Only two studies [46, 92] synthesized a 3D MRI volume, as shown in Tables 2, 3, 4 and 5.

## Conclusion

Deep generative modeling is a key technology for alleviating important limiting factors that render data collection challenging, such as the natural prevalence of several cancer types, morphological diversity of lesions and lack of standardization of MRI protocols. Although there are some trustworthiness issues in many of the presented studies, we argue that when implemented properly by strictly following the corresponding best practices and recent advances in this field, generative models have the potential to revolutionize medicine by correcting the class imbalances of the disease in the dataset, diversifying anatomically the available region of interest, providing vendor-specific samples and supporting downstream tasks with larger training sets.

## Abbreviations

ADC: Apparent diffusion coefficient
ADNI: Alzheimer’s disease neuroimaging initiative
AI: Artificial intelligence
ANN: Artificial neural network
APTw: Amide proton transfer weighted
AsynDGAN: Asynchronized discriminator generative adversarial network
BraTS: Multimodal brain tumor segmentation
Cap-GAN: Capsule network-based generative adversarial network
CB-GAN: Coarse-to-fine boundary aware generative adversarial network
CG-SAMR: Confidence-guided synthesis of anatomic and molecular MRI images network
cGANe: Constrained generative adversarial network ensembles
CNN: Convolutional neural network
CovNets: Convolutional networks
cGANs: Conditional generative adversarial networks
CS: Clinical significance
CT: Computerized tomography
DSC: Mean of dice similarity coefficient
DC-AL GAN: Deep convolutional ALexNet generative adversarial network
DCE: Dynamic contrast-enhanced
DCGAN: Deep convolutional generative adversarial network
DL: Deep learning
DTI: Diffusion tensor imaging
Enh-Seg-GAN: Enhancement and segmentation generative adversarial network
FD: Frechet distance
FLAIR: Fluid-attenuated inversion recovery
GANs: Generative adversarial networks
IDH1: Isocitrate dehydrogenase 1
JSD: Jensen-Shannon divergence
KLD: Kullback–Leibler divergence
LENet: Laplacian eigenmaps network
MAE: Mean absolute error
MM-GAN: Manifold matching generative adversarial network
mp-MRI: Multi-parametric magnetic resonance imaging
MRI: Magnetic resonance imaging
MSE: Mean squared error
MSG-GAN: Multi-scale gradient generative adversarial network
MUNIT: Multimodal unsupervised image-to-image translation
NIN: Network-in-network
PCA: Principal component analysis
PCa: Prostate cancer
PGGANs: Progressive growing of generative adversarial networks
pNET: Pancreatic neuroendocrine tumor
PPWGAN-GP: Preserving-adversarial network
PRISMA: Preferred Reporting Items for Reviews and Meta-Analysis
PSNR: Peak signal-to-noise ratio
PsP: Pseudoprogression
ResNet: Residual network
ROI: Regions of interest
SAG-GAN: Semi-supervised attention-guided generative adversarial network
SAMR: Synthesis of anatomic and molecular MRI images network
SMIG: Synthetic medical image generator
SPADE-GAN: Spatially adaptive (de)normalization generative adversarial network
SVM: Support vector machine
SSIM: Structural similarity index measure
T1w: T1-weighted
TGP: Tumor growth predictor
T2w: T2-weighted
T1ce: T1 contrast-enhanced
t-SNE: T-distributed stochastic neighbor embedding
TTP: True tumor progression
UCG-SAMR: Unsupervised confidence-guided synthesis of anatomic and molecular MRI images network
VAE: Variational autoencoders
VALF: Variation-aware federated learning framework
WGAN: Wasserstein generative adversarial network
WHO: World Health Organization
